# Relative age in the school year and risk of mental health problems in childhood, adolescence, and young adulthood

**DOI:** 10.1111/jcpp.13684

**Published:** 2022-08-15

**Authors:** Thomas Broughton, Kate Langley, Kate Tilling, Stephan Collishaw

**Affiliations:** 1 Division of Psychological Medicine and Clinical Neurosciences, MRC Centre for Neuropsychiatric Genetics and Genomics, School of Medicine, Cardiff University, Cardiff, UK; 2 Wolfson Centre for Young People’s Mental Health, Cardiff University, Cardiff, UK; 3 School of Psychology, Cardiff University, Cardiff, UK; 4 MRC Integrative Epidemiology Unit, University of Bristol, Bristol, UK

**Keywords:** Relative age, Avon Longitudinal Study of Parents and Children (ALSPAC), Depression, Mental Health

## Abstract

**Purpose:**

Relative age within the school year (“relative age”) is associated with increased rates of symptoms and diagnoses of mental health disorders, including ADHD. We aimed to investigate how relative age influences mental health and behaviour before, during, and after school (age range: 4-25 years).

**Method:**

We used a regression discontinuity design to examine the effect of relative age on risk of mental health problems using data from a large UK population-based cohort (Avon Longitudinal Study of Parents and Children (ALSPAC); N=14643). We compared risk of mental health problems between ages 4 and 25 years using the parent-rated Strengths and Difficulties Questionnaire (SDQ), and depression using self-rated and parent-rated Short Mood and Feelings Questionnaire (SMFQ) by relative age.

**Results:**

The youngest children in the school year have greater parent-rated risk of mental health problems, measured using parent-rated SDQ total difficulties scores. We found no evidence of differences before school entry

(estimated standardized mean difference (SMD) between those born on 31^st^ August and 1^st^ September: 0.02 [-0.05, 0.08]).

We found that estimates of effect size for a one-year difference in relative age were greatest at eleven years (SMD: 0.22 [0.15, 0.29]), but attenuated to the null at twenty-five years (SMD: -0.02 [-0.11, 0.07]). We did not find consistent evidence of differences in self-rated and parent-rated depression by relative age.

**Conclusion:**

Younger relative age is associated with poorer parent-rated general mental health, but not symptoms of depression.

## Introduction

Up to one in eight children in the United Kingdom meet diagnostic criteria for mental health disorders (Sadler et al., 2018). Mental health disorders have become more prevalent in children over time and are associated with immediate and long-term physical and psychological impairment (Sadler et al., 2018; Vos et al., 2017). Policy and practice interventions targeting potentially modifiable risk and protective factors will only be effective if the risk factors are truly causal (Thapar & Rutter, 2019). In observational studies, it is hard to infer causality due to the possibility of unmeasured confounding (Thapar & Rutter, 2019). To overcome this, we can use ‘natural experiments’ which approximate the random assignment of risk or protective conditions. (Thapar & Rutter, 2019).

One potential risk factor is a child’s age within the school year, henceforth, relative age. In England and Wales, this is determined by the cut-off date for school entry of the 1^st^ of September. Children born in September are the oldest in the school year, and those born in August the youngest. This leads to an age difference of up to twelve months between children in the same class; at school entry, it is assumed that the oldest children will be approximately 20% more mature than the youngest children in the school year (Holland & Sayal, 2019). Relative age in England and Wales is assigned quasi-randomly since one cannot directly influence a child’s exact delivery date (assuming natural birth), even though children’s dates of birth are unevenly distributed across the school year with a higher birth rate in late September (Borja & Martin, 2017). Potential confounders of mental health outcomes would be plausibly independent of birth being either side of the school entry cut-off within a narrow timeframe around that date, with this assumption becoming less plausible as the bandwidth increases. The assumption that being born either side of the cut-off is randomly allocated cannot be tested, but its association with observed confounders can be. Thus, observational studies of relative age allow for causal inference, using regression discontinuity methods where there is a continuous variable (day of year born) which has a cut-off (1^st^ of September) at which the treatment (entry to school) is applied (Moscoe, Bor, & Bärnighausen, 2015; Thistlethwaite & Campbell, 1960).

The youngest children in the school year are over-represented in mental health disorder statistics (Root et al., 2019). Children born in the last quarter of an academic year were approximately 23% more likely to be diagnosed with depression than those born in the first quarter (Root et al., 2019). It is unclear whether this represents a real difference in disorder risk by relative age, or whether this is due to differences in help-seeking patterns or by referrals to mental health services.

Relative age effects on mental health are relatively unexplored from an epidemiological perspective. Three UK studies suggest that the youngest children in the school year have higher parent, teacher, and self-rated mental health symptom scores (Crawford, Dearden, & Greaves, 2013; Patalay et al., 2015, Norbury et al., 2016). Crawford et al (2013) compared teacher and parent-rated versions of the Strengths and Difficulties Questionnaire (SDQ, Goodman, 1997) in seven- to thirteen-year-old children in the Avon Longitudinal Study of Parents and Children (ALSPAC) cohort. Children born in August had greater total SDQ scores relative to their September-born peers at age seven. The researchers also found that relative age effects on mental health attenuate to the null as the gap between oldest and youngest children becomes proportionally smaller as chronological age increases (Crawford et al., 2013). Patalay et al (2015) investigated self-rated SDQ scores in eleven- to thirteen-year-old children in England, grouped into thirds by relative age. The youngest third of children had higher SDQ scores, primarily driven by emotional and peer problems. Lastly, Norbury et al (2016) found that the youngest children in their first year of compulsory education were at higher risk of behaviour problems as evidenced by higher SDQ scores, as well as poorer language skills, relative to their peers (Norbury et al., 2016)

Cross-national comparisons of large representative population surveys of mental health disorders have shown that being born in the latest third of any academic year was associated with an increased risk of depression diagnosis, as well as increased self-rated, parent-rated, and teacher-rated risk of mental health problems (Goodman, Gledhill, & Ford, 2003). Relative age effects were present across nations with different school entry cut-off dates, for example, in England (September cut-off), Scotland (March cut-off), and Northern Ireland (July cut-off). The same study found that relatively young children did not differ from older peers on age-standardised ability tests (Goodman et al., 2003). This evidence shows that relative age effects are independent of other birth date effects such as season of birth, and that younger children are more likely to have a greater risk of mental health problems regardless of rater, chronological age, and a country’s school entry cut-off.

There are several important knowledge gaps. First, it is not known whether differences in mental health by relative age occur prior to starting school. Typically, before school entry children are not grouped together by the school entry cut-off; if mental health differences by relative age emerge after, but not before, school entry, we can infer that these differences are caused by this grouping.

Second, it is not known whether relative age effects extend into adulthood. To get a clearer picture of relative age effects and potential interventions for these effects, it is not only important to consider what happens when relatively young children enter compulsory education, but also when they leave. Third, psychometric tests, such as the SDQ show different rates of mental health problem risk, and different patterns of associations with a range of risk factors, depending on the informant used - parent, young person or teacher (Collishaw, Goodman, Ford, Rabe-Hesketh, & Pickles, 2009). This emphasises the need for multi-informant sources of risk of mental health problems. Lastly, most previous studies have looked at general mental health symptom screens such as the SDQ. However, these studies highlight some specificity across different domains of mental health (Patalay et al., 2015). To better understand mental health domain-specific effects, more sensitive and specific measurements of particular mental health outcomes are needed, such as the Mood and Feelings questionnaire (MFQ; (Angold et al., 1995) for depression.

This study aims to investigate how relative age influences mental health before, during, and after school, using data from a large UK population-based cohort (ALSPAC) repeatedly assessed between the ages of 4 and 25 years. We hypothesise that the youngest children in the school year will have greater reported risk of mental health problems than their relatively older peers, that relative age differences will first emerge at school entry, and these differences will be strongest in the early school years. This is because the difference in chronological age (and therefore physiological and psychological maturity) is greatest between oldest and youngest in those years. We hypothesised that there would be no difference by relative age before school entry because we assumed that pre-school children are largely taught through play, and so are not usually subject to formal assessments and classroom streaming to the same degree as children who have entered the school system.

## Method

### Sample

The Avon Longitudinal Study of Parents and Children (ALSPAC) is an ongoing prospective longitudinal birth cohort of individuals born in the historical Avon administrative county in South-West England (Boyd et al., 2013; Fraser et al., 2013; Northstone et al., 2019). Pregnant women resident in Avon, UK with expected dates of delivery 1/4/1991 to 31/12/1992 were invited to take part in the study. The initial number of pregnancies enrolled is 14,541 resulting in 14,062 live births and 13,988 children alive at 1 year of age (Boyd et al., 2013).

When the oldest children in the cohort were approximately 7 years of age, the initial sample was bolstered with 913 eligible children who did not initially join the study. The total sample size for analyses using any data collected after the age of seven is therefore 15,454 pregnancies, resulting in 15,589 foetuses. Of these 14,901 were alive at 1 year of age.

The ALSPAC study website contains details of all available data, available through a fully searchable data dictionary and variable search tool http://www.bristol.ac.uk/alspac/researchers/our-data/). Study data gathered from participants at 22 years and onwards were collected and managed using REDCap electronic data capture tools hosted at the University of Bristol (Harris et al., 2009). REDCap (Research Electronic Data Capture) is a secure, web-based software platform designed to support data capture for research studies.

Individuals were included in the current study when data were available on week, month, and year of birth, together with their school year at any given assessment age; participants were excluded if they were not alive at one year of age (N=688), were not in the expected school year given their chronological age (N=12), and if they were the younger of a twin pair (N=186). After excluding these participants, 14,643 individuals were included in the analyses.

### Ethical Considerations

Ethical approval for the study was obtained from the ALSPAC Ethics and Law Committee and the Local Research Ethics Committees. Informed consent for the use of data collected via questionnaires and clinics was obtained from participating families following the recommendations of the ALSPAC Ethics and Law Committee at the time.

### Relative age

Children’s exact birth dates were not provided due to the risk of deanonymization. Children’s birth dates were grouped into one eight-day block (1^st^-8^th^ September) and fifty-one consecutive 7-day blocks ending on August 31^st^. Children were assigned a score reflecting week of birth relative to the academic year (range 0 (Oldest; 1^st^ September) − 51(Youngest, up to 31^st^ August)).

### Child mental health

**Figure 1:**
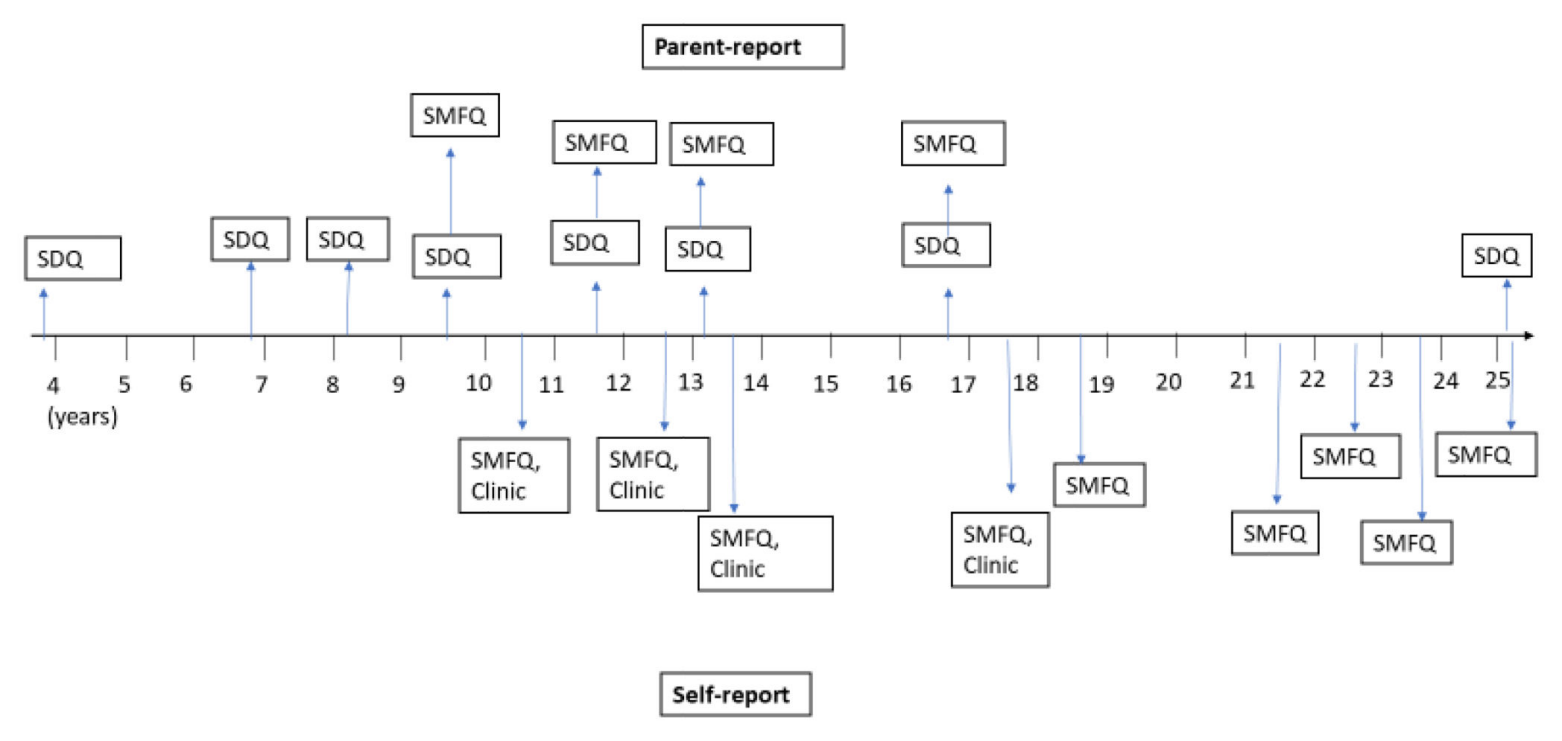
Timeline of ALSPAC Mental Health Measures

Two well-validated child and adolescent mental health screening questionnaires were administered at several timepoints between 4-25 years; the Strengths and Difficulties Questionnaire (SDQ; Goodman, 1997) and the Short Mood and Feelings Questionnaire (SMFQ; Angold et al., 1995). We chose to use SDQ and SMFQ data continuously for statistical (increased power) and conceptual reasons. Previous research suggests that mental health and neurodevelopmental conditions such as depression and ADHD lie at the end of a continuous distribution of underlying symptom traits, with similar aetiological and outcome profiles (Thapar, 2018; Thapar, Collishaw, Pine, & Thapar, 2012). Figure 1 shows the timepoints for administration. We present further details of these questionnaires in the supplementary materials (S1).

### Covariates

An advantage of regression discontinuity methods is that they do not rely on controlling for confounders as in usual observational studies (Oldenburg, Moscoe, & Bärnighausen, 2016), it is still important to test assumption violations such as unbalanced covariate distribution. We included various early pre-assignment covariates:

Maternal background: Age of mother at birth, mother’s education (highest qualification), maternal depression at 18 weeks gestation (Edinburgh Postnatal Depression Scale; (Cox, Holden, & Sagovsky, 1987).

Pregnancy and birth: Caesarean birth, birth size (i.e., single, or multiple birth), birthweight, gestational age, maternal alcohol use during last two months of pregnancy, maternal smoking during pregnancy.

Child factors: household crowding, child ethnic background, home ownership, parity, Sex. We controlled for age of child at questionnaire completion (in months, to nearest month). The questionnaires were sent out to parents to map on to certain timepoints throughout development. The age at which questionnaires was filled in had a wide spread across participants, and was not different between August born and September born children (see table S2) For further information relating to children’s ages and questionnaire timepoints, we include a table in the supplementary materials (table s2).

### Design

We used a regression discontinuity design to compare the relative risk of mental health problems by relative age (Hilton Boon, Craig, Thomson, Campbell, & Moore, 2021; Moscoe et al., 2015; Oldenburg et al., 2016; Venkataramani, Bor, & Jena, 2016). The exposure is “age at starting school” and the running variable is “week of birth”, with the discontinuity on 1^st^ September. We assumed a “sharp” RD design given the strict cut-off date for school year selection in England and Wales; we assumed (based on LEA rules at the time) that children started school at the same time and during the school year they turn 5 years old, and that schools and LEAs adhere to this cut-off. Regression discontinuity relies on the assumptions that:

1: The decision rule and cut-off value are known
2: There are no confounders of age at starting school and outcome that are related to the cut-off
3: The outcome (risk of mental health problems) would not show a discontinuity at the threshold in the absence of the exposure (being admitted to school) (Moscoe et al., 2015).

### Statistical Analysis

We checked whether regression discontinuity design assumptions were met by comparing covariate distributions across the school year to ensure that the cut-off is not associated with any other variables besides relative age. We checked distributions of maternal depression, Maternal age, gestation, birthweight, birth size, pre-natal alcohol use, smoking, caesarean status, crowding, home ownership status, mother’s education, and parity across the cut-off. We also checked the distribution of births across the months of the school year. We carried out the analysis in three ways; first, one where the exposure is continuous throughout the school year and there is no selection window near the cut-off (henceforth, “no bandwidth”), second, restricted to those born four weeks either side of the September 1^st^ cut-off (“4 weeks), and lastly, restricted to those born 8 weeks either side of the cut-off (“8 weeks”). The four-week and eight-week bandwidths only compare those with birthdates within those time windows around September 1st. We chose these bandwidths because in narrower bandwidths (e.g., 4 weeks) the assumption of no confounding is more plausible. We viewed the estimation of effects of relative age as local randomness near the cut-off, by limiting analysis to observations that lie within one bandwidth on either side of the 1^st^ September cut-off (i.e. four weeks either side of the cut-off, in the case of a four-week bandwidth). After bandwidth selection, we fit local linear regressions on observations within the bandwidth to estimate the effect of relative age.

Previous studies have used similar bandwidths (Crawford et al., 2014). A bandwidth of 8 weeks corresponds to approximately the length of a school half-term. A problem with using longitudinal cohort data is participant attrition over time; at age 4 years there were 9312 participants, decreasing to 4076 at 25 years. we compared complete cases with those who had incomplete data and then we used multiple imputation by chained equations (see supplementary 3 and 4 for further information) to account for selective attrition. We included maternal depression, maternal age, gestation, birthweight, birth size, alcohol use in last 2 months of pregnancy, smoking, caesarean status, crowding, home ownership status, mother’s education, and parity in the imputation models. We imputed to a maximum sample of those with at least one measure for each mental health outcome (N SDQ = 11116; N Self SMFQ = 9468; N Parent SMFQ = 9146). We then used linear regressions for each outcome on age at starting school, then also adjusted for all abovementioned covariates. We checked all models for Monte Carlo error following guidelines (White, Royston, & Wood, 2011), further details in the supplementary (S3; S4).

To test robustness of findings, we also report findings from a complete-case analysis at each outcome point.

We also used a generalized estimating equation (GEE) approach to model our outcomes (parent-rated SDQ, self-rated SMFQ, and parent-rated SMFQ) by relative age, and other covariates, as a sensitivity analysis. The advantages of this approach are that GEE models have some robustness to attrition, but do not use imputation (i.e. only analyse available data)(Liang & Zeger, 1986).

Secondary analyses tested SDQ subscales by relative age, and potential interactions by child sex. All analyses were conducted using Stata (v16.1 SE, StataCorp LLC, College Station, TX).

## Results

### Testing assumptions of the regression discontinuity design − covariate pattern by month of birth

Supplementary tables S5 and S6 display demographic characteristics. First, we found similar patterns of covariate distribution between children born August vs September, showing no discontinuous relationship between ‘pre-treatment’ covariates and relative age. Second, we found a similar distribution of births in the study sample across the months of the year (Graph S7), so we found no evidence of a discontinuity in distribution of births around September 1st. Lastly, we found similar patterns of missing data (tables S8-S13) Thus, we found no evidence that the regression discontinuity design assumptions, listed above, were violated.

### Comparison of participants with complete vs incomplete data

Participants with complete records at all ages and assessments were more likely to be female, first-born, white, with parents who are older, non-smokers, higher educated and less depressed than those with incomplete data (table S14). This suggests that a complete-case analysis approach would be biased (Lee et al., 2021).

### Descriptive data on mental health outcome measures

Table S15 presents descriptive, unstandardised data on mental health outcome variables for the whole sample (with available data at a given time point), and for children born in August and September. Table S16 presents the same data for individuals included in the 4-week and 8-week bandwidths. For each measure, higher scores indicate greater reported SDQ scores, which are indicative of greater risk of mental health problems

### Relative age

**Figure 2:**
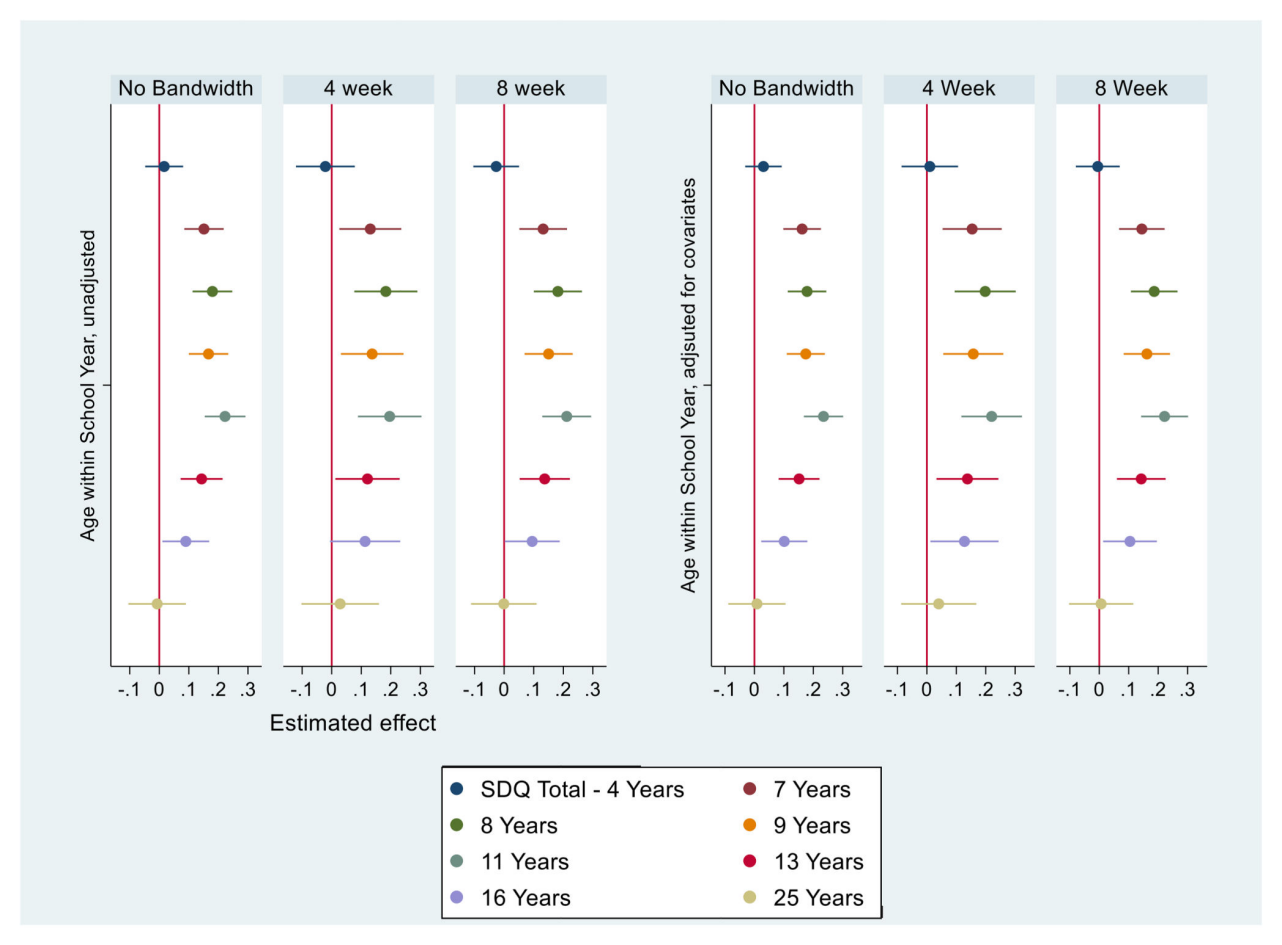
Parent-rated Mean Standardized SDQ Total Difficulties Coefficient Plots, Unadjusted Models (left), models adjusted for covariates (right), Imputed data. “Estimated effect” represents SMD by relative age. “No Bandwidth” = All participants included; “4 Week” = Restricted to participants born up to 4 weeks either side of September 1^st^ Cut-off; “8 week” = Restricted to participants born 8 weeks either side of the September 1^st^ cut-off. N=11116

As shown in Figure 2 (Parent-rated general mental health) we found no evidence of an effect of relative age on parent-rated mental health before entry into school (age 4 Standardised Mean Difference (SMD): 0.02, 95% CI: [-0.05, 0.08]) At the earliest point after school entry (7 years) we found that a 1-year decrease in relative age in the school year was associated with a difference of approximately one-sixth of a standard deviation in SDQ total difficulties (SMD: 0.15, 95% CI: [0.08, 0.22]). Being relatively young in the school year was associated with higher SDQ total difficulty scores, indicative of poorer parent-rated child mental health. These differences persisted throughout the school years, with the strongest effect at eleven years (SMD: 0.22, 95% CI: [0.15, 0.29]). These differences attenuated to the null at 25 years (SMD: -0.01, 95% CI: [-0.1, 0.09]). Results were materially unchanged after adjusting for covariates.

**Figure 3:**
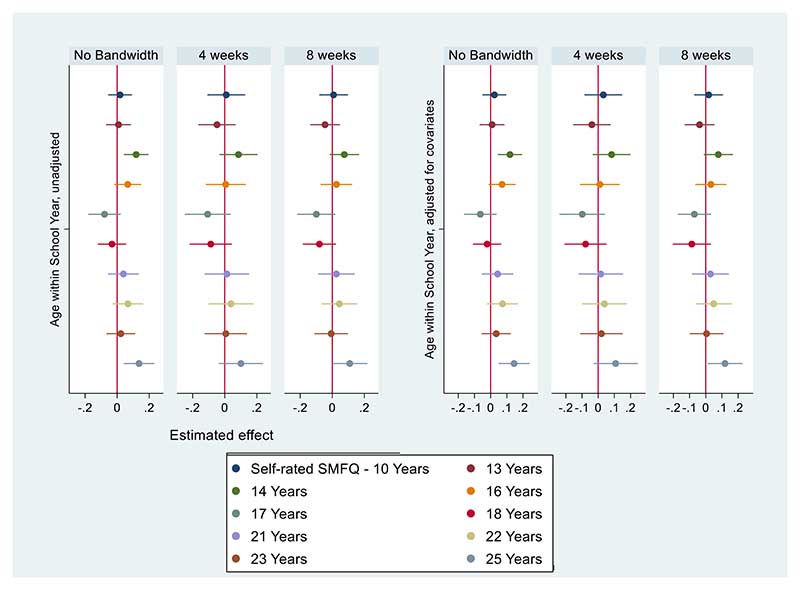
Self-rated SMFQ Coefficient Plots - Unadjusted Models (left), models adjusted for covariates (right). Imputed data. Estimated effect represents SMD by relative age. N=9468

As shown in Figure 3 (self-rated depression symptoms) we found that younger children in the school year are more likely to report self-rated depression symptoms at fourteen years (SMD: 0.12, 95% CI: [0.04, 0.20]) and at twenty-five years of age (SMD: 0.14, 95% CI: [0.04, 0.23]), but there was no evidence of difference by relative age at other ages.

**Figure 4:**
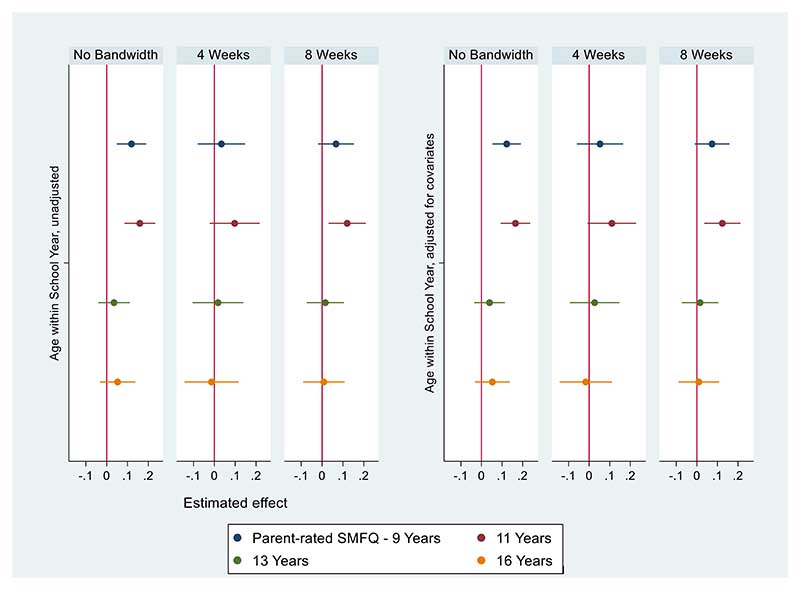
Parent-rated SMFQ coefficient plots - Unadjusted Models (left), models adjusted for covariates (right). Imputed data. Estimated effect represents SMD by relative age. N=9146

Figure 4 (parent-rated depression) shows that relatively young children scored higher in parent-rated depression at nine years (SMD: 0.12, 95% CI: [0.05, 0.19]) and eleven years (SMD: 0.16, 95% CI: [0.09, 0.23]). We found no other evidence of differences by relative age on parent-rated SMFQ scores.

Regression tables of our findings are also available as supplementary material (Tables S17-S25)

### Sensitivity Analysis

We found that most of the identified relative age effects on parent-rated SDQ scores are materially unchanged when restricted to children born 4 weeks either side of the cut-off, except for the association at sixteen years attenuating to the null. Additionally, restricting the bandwidths to those born in the months closest to the cut-off yielded wider 95% confidence intervals. For the self-rated SMFQ, we found no evidence of differences within the 4-week bandwidth, except at 25 years.

The complete-case results resembled the analysis using multiple imputation, with some differences. We present these findings in tables S26-S28. Complete-case analysis identified effects of relative age on parent-rated SMFQ scores at 16 years (SMD: 0.10 [0.01, 0.20]); these attenuated to the null in the multiple imputation analysis. The multiple imputation analysis showed an effect of relative age on self-rated SMFQ scores at 25 years (SMD: 0.16 [0.06, 0.25], in contrast to complete-case analyses (SMD: 0.09 [-0.02, 0.20], albeit with overlapping confidence intervals.

As an alternative approach to multiple imputation, we produced General Estimating Equations (GEE) models of relative age effects on all outcomes. We found that the GEE models strongly support the models using imputed data (Tables S29-S31).

## Secondary Analyses

### SDQ Subscales

We explored SDQ subscale scores by relative age. All subscale regression model information is provided in Table S17. We found that the hyperactivity subscale followed the same pattern as the total difficulties scores, in addition to showing the largest SMDs by relative age of all subscales, up to.25 of a standard deviation at age 11 years [95%CI:.18,.32]. Parent-rated emotional problems and peer problems also showed effects by relative age in the same direction (SMD emotional at 7 years:.13; [95%CI:.06,.20]; SMD peer at 11 years;.15; [95%CI:.08;.22]. We did not find consistent relative age differences for conduct problems.

### Interactions by sex

We found no consistent difference in relative age effect sizes for males and females, for the SDQ and both versions of the SMFQ (available on request).

## Discussion

The present study aimed to investigate the effect of relative age in the school year on risk of mental health problems in a general population longitudinal cohort studied across childhood, adolescence and into young adulthood. Using the fact that relative age corresponds to a regression discontinuity design, we found that the youngest children in the academic year have greater parent-rated general risk of mental health problems, and that these findings were materially unchanged after adjusting for covariates. We found that the hyperactivity subscale accounts for the largest differences by relative age, with emotional problems and peer problems also contributing. Further, the findings support our prior hypothesis that relative age differences are only present after school entry.

We also found that relative age effects persist into secondary education, but wane after young people leave school. The largest differences by relative age were at eleven years. This differs from our prior hypothesis that relative age differences are strongest at the earliest measurement after school entry, and then subsequently weaken as children develop, on the basis that the difference of up to one year between children becomes increasingly less noticeable by chronological age. The transition to secondary school appears to be a particular period of vulnerability for relatively young children. We suggest that relative age effects attenuate to the null by adulthood as the gap between oldest and youngest in the year becomes proportionately less as children age chronologically. The gap between the oldest and youngest is up to one year. At the beginning of school, ages four to five years, the oldest children will be approximately 20% more psychologically and physiologically mature than a child born in August. By age sixteen, this is reduced to 6.25%. We also expected to find relative age effects on specific measures of depression. However, while we did find some differences between the oldest and youngest children, we found no consistent pattern of effects across development; the majority of the self-rated and parent-rated SMFQ measurements showed no meaningful relationship between relative age and depression symptoms.

Investigating potential mechanisms through which relative age affects mental health was beyond the scope of the present study, but previous research shows that younger children in the school year have poorer education attainment and are more likely to be bullied (Crawford et al., 2013; Mühlenweg, 2010). Both are associated with increased risk of mental health problems in children and young people (Klomek, Sourander, & Elonheimo, 2015). Therefore, a further question for research to consider is whether bullying influences the relationship between relative age and mental health. A second question for further research is whether pubertal changes influence this relationship (Copeland, Worthman, Shanahan, Costello, & Angold, 2019). A third suggestion is exploring whether relative age has differential effects on anxiety vs depression symptoms. We found relative age effects on the parent-rated SDQ emotional symptom subscale, which includes both anxiety and depression symptoms, but found no consistent effects for the SMFQ, which specifically measures depressive symptom traits. Fourth, research shows that the youngest children in the school year are perceived to have less developed language skills relative to older peers (Norbury et al., 2016). Further research is needed on whether language development mediates relative age effects on risk of mental health problems.

Lastly, identifying relative age effects in more vulnerable groups, such as children with neurodevelopmental disorders (Addicoat, Thapar, Riglin, Thapar, & Collishaw, 2019) and exploring the extent to which neurodevelopmental disorder traits moderate the relationship between relative age and mental health would be an interesting direction for future research. This is because the hyperactivity subscale, a measure of ADHD symptoms, showed the strongest relative age effects in the analysis. Relatively young children are more likely to be diagnosed with neurodevelopmental disorders such as ADHD (Root et al., 2019), but it is not known whether relative age effects are especially pronounced among children with neurodevelopmental disorders, and whether children with neurodevelopmental disorders who are relatively young constitute a particularly high-risk group that would warrant additional support as they start school. We used the same measure (the SDQ) across development, using the same rater (the parent). Evidence supports the reliability and validity of the SDQ as a measure of mental health in children and adolescents (Goodman, 1997; 2001) and emerging evidence supports its use in young adulthood (Riglin et al 2021). However, further evidence is needed assessing measurement invariance of the SDQ and its subscales across the age range covered by this study.

Our findings support Patalay et al.’s (2015) findings that relative age influences emotional problems in children, and Crawford et al.’s findings that relatively young children show poorer social and emotional development as reflected by poorer outcomes on the SDQ. However, Crawford et al. (2013) found that parent-rated differences in SDQ scores by relative age are not present beyond the age of nine years in the ALSPAC cohort, whereas in the same cohort we find that relative age effects persist up to the age of sixteen years. However, in both studies, estimates were in the same direction. In contrast to Crawford et al, we used varying approaches to account for participant attrition (which became more pronounced as children grew older) and a more precise measure of relative age (in weeks). The two studies also differ in the covariates included.

The present study adds to previous findings by investigating the relationship between relative age and mental health in the ALSPAC cohort both prior to and during school age, as well as extending to after young people they have left school. We also add to previous findings by testing relative age effects on a specific measure of depression symptom traits (SMFQ), and by using week of birth as a more precise measurement of age within school year. We used imputed data and longitudinal analyses to attempt to control for biases that may arise from missing data resulting from attrition in the ALSPAC cohort, and we used alternative selection bandwidths as sensitivity analyses. The strengths of the study are that we used rich data from a single longitudinal population cohort with data collected throughout development, and the study used consistent and widely implemented multi-informant measures of mental health. We checked for violations of the RD design, there was no evidence that covariates act as confounders because there was no inequality in distribution across the school entry cut-off. Therefore, this is suggestive of a causal effect of young relative age on mental health in school age children. We are confident that our findings may represent causality given that regression discontinuity approaches can estimate causal effects of “treatment” (i.e. being relatively old for the school year), when other experimental methods such as randomized controlled trials are not feasible (Moscoe et al., 2015; Venkataramani et al., 2016). A causal interpretation relies on the assumption that, apart from relative age, individuals born on either side of the September 1^st^ cut-off rule, but close to it, are similar in other characteristics that are not affected by this rule. If this assumption is not violated, then we can infer that individuals close to the cut-off are effectively randomly selected for “treatment”. We have demonstrated that there is no evidence that the assumption does not hold in the present study (see table S5). Therefore, we can be confident that the increase in risk of mental health problems in the relatively youngest individuals was the result of being young in the school year.

This study has limitations. First, data missingness due to participant attrition may result in bias due to differences between participants who are retained vs drop out. We attempted to mitigate this by using a multiple imputation approach to missing data, however, if children at greater risk of mental health problems are more likely to drop out of the study, the use of multiple imputation will not remove all the bias (Lee et al., 2021). The pattern of findings was also the same when using GEE models − a different method for dealing with attrition. Second, there was a long gap between some measurements, including a nine-year gap between the last two SDQ measurements, so we did not know precisely when effects attenuated to the null.

Third, we did not consider participants’ childcare arrangements before school entry; some children may have experienced more formal pre-school settings, and we did not test if effects differed compared to children without these childcare arrangements. Similarly, we did not consider the type of schooling children entered, and whether classes were comprised of single or multiple academic year groups taught together. Lastly, we did not know exactly when the children started school, which would lead to a “fuzzy” regression discontinuity design. In the UK, education authorities often offer some flexibility about when in the calendar year children start their first year of school (e.g. at the start of the autumn or spring terms) with a view to helping ensure school readiness including amongst summer born children. Nevertheless, summer born children typically remain the youngest in their school year, and further research is needed to assess whether there are impacts of delayed school entry on children’s mental health.

### Implications

The effect of relative age on risk of mental health problems is modest at an individual level but may be larger at a population level. It appears that the effects of relative age may specifically impact children of school age, therefore, any intervention should be applied at this period. Relative age effects may attenuate to the null by adulthood, but evidence suggests there are links with broader psychosocial outcomes, such as educational attainment and employment (Lopez-Lopez et al., 2019)

Previous authors have suggested school admissions and entry system changes, including delaying school entry (Dee & Sievertsen, 2018) or age-based assessment adjustments (Crawford et al., 2013). Changes to established school structures and admission systems may be difficult to implement in practice because of the likelihood of increased disruption for schools, teachers, and families. Evidence is mixed on the benefits of this practice to children (Dhuey, Figlio, Karbownik, & Roth, 2019), and more evidence is required on whether age adjustments to grades and examinations counteract relative age effects. It is important to remember that in any classroom some children will be younger than others, even when combining more than one year group together. We are cautious about suggesting what the effect of relative age on clinical aspects of mental health problem risk looks like in practice, given the modest size of these effects, and that we did not consider risk of clinically diagnosed mental health problems. Being young for the school year may not affect individuals equally, and it is important to further investigate relative age effects on mental health using measures that consider clinical impacts both in terms of presentation of symptoms and their impacts on functioning.

An alternative approach may involve organising the school register by age within school year to raise awareness of who is relatively young within the classroom (Norbury et al., 2016) which may facilitate differentiated instruction and assessment. We suggest that schools become more aware of relative age effects on education achievement and mental health on their pupils.

## Conclusion

The present study provides a long-term longitudinal follow-up examining relative age effects across development using consistent measures of mental health. The study assumptions of the regression discontinuity design were examined for plausibility and adjusted for observed covariates, suggesting a causal relationship between relative age and mental health. We found that younger children in the school year have higher parent-rated general risk of mental health problems compared to their older peers. Young relative age may be a potentially modifiable causal factor for child and adolescent risk of mental health problems, but we did not consistently find the same effect for depression.

## Supplementary Material

Supplementary

## Data Availability

The informed consent obtained from ALSPAC participants does not allow for the data to be made freely available through any third party maintained public repository. However, data used for this submission can be made available on request to the ALSPAC Executive. The ALSPAC data management plan describes in detail the policy regarding data sharing, which is through a system of managed open access. Full instructions for applying for data access can be found here: http://www.bristol.ac.uk/alspac/researchers/access/. The ALSPAC study website contains details of all the data that are available (http://www.bristol.ac.uk/alspac/researchers/our-data/)
