# Supplementary material for: Relative age in the school year and risk of mental health problems in childhood, adolescence and young adulthood
Source: J Child Psychol Psychiatry. 2022 Aug 15;64(1):185–96. doi: 10.1111/jcpp.13684 (PMC7613948; doi:10.1111/jcpp.13684)
Supplement: Supplementary file 1 — Appendix S1. Questionnaires used to measure risk of mental health problems in the present study. Appendix S2. Multiple imputation. Table S1. Table summary of ages of ALSPAC participants at completion of questionnaires. Note IQR = Interquartile Range. Table S2. Multiple Imputation variables, models used, and percentage of data missing from these variables/models. Table S3. Demographic characteristics of the study sample. Table S4. Demographic information for participants born 4 (“4 weeks”) and 8 (“8 weeks”) weeks either side of the September 1st Cut‐off. Table S5. Missing table patterns of SDQ total difficulties. Table S6. Missing table patterns of SDQ total difficulties. Table S7. Missing table patterns of self‐rated SMFQ. Table S8. Missing table patterns of self‐rated SMFQ. Table S9. Missing table patterns of parent rated SMFQ. Table S10. Missing table patterns of parent rated SMFQ. Table S11. Logistic regressions of covariates on being a complete‐case (i.e., having all data at all mental health timepoints). Table S12. Descriptive statistics of outcome measures in August and September born children (Parent‐rated SDQ Total Difficulties, self‐rated and parent‐rated SMFQ, unstandardized, complete‐case data). Table S13. Descriptive statistics of outcome measures (Parent‐rated SDQ Total Difficulties, self‐rated and parent‐rated SMFQ, unstandardized, complete‐case data; restricted to 4 week bandwidth (left) and 8 week bandwidth (right)). Table S14. Regression results for SDQ total difficulties and subscales by relative age, Imputed data (N = 11,116). Table S15. Regression results for Self‐rated SMFQ by relative age, Imputed data (N = 9,468). Table S16. Regression results for parent‐rated SMFQ by relative age, Imputed data (N = 9,164). Table S17. Regression results for parent‐rated SDQ total difficulties scores by relative age, restricted to 4 weeks either side of September 1st cut‐off, Imputed data (N = 2,035). Table S18. Regression results for Self‐rated SMFQ by relativ [file JCPP-64-185-s001.docx]

**Supporting Information**

**Appendix S1.** Questionnaires used to measure risk of mental health problems in the present study.

*Strengths and Difficulties Questionnaire:*

The parent-report Strengths and Difficulties Questionnaire (SDQ; (Goodman, 1997) is a brief emotional and behavioural screening questionnaire validated in children and young adults (Cronbach alpha .73, test–retest reliability: .62) (Goodman, 2001; Riglin et al., 2021). There are twenty-five items in the SDQ, with five subscales each comprised of five items assessing emotional problems, conduct problems, peer problems, hyperactivity and inattention problems, and prosocial behaviours. For each question, the answer can be “not true” (scored as 0), “somewhat true” (scored as 1) and “certainly true” (scored as 2), with some items (e.g., ‘sees tasks through, good attention span’) reverse coded so that a higher score indicates greater difficulty. Subscale scores range from 0-10, and SDQ difficulties from the first four subscales are summed to provide a total difficulties score measuring overall risk of mental health problems (range 0-40).

*Short Mood and Feelings Questionnaire*

For depression, we used parent-rated and self-rated versions of the short mood and feelings questionnaire (SMFQ, (Angold et al., 1995), a brief (13-item), validated measure of depression symptoms over the preceding two-week period (Angold et al., 1995; Eyre et al., 2021) For each question, the answer can be “not true” (0), “sometimes true” (1) and “true” (2). The SMFQ has a range of between 0 and 26; higher scores indicate more severe depressive symptoms. The SMFQ shows good reliability and internal consistency (Angold et al., 1995; Turner, Joinson, Peters, Wiles, & Lewis, 2014).

References

Angold, A., Costello, E., Messer, S., Pickles, A., Winder, F., & Silver, D. (1995). The development of a short questionnaire for use in epidemiological studies of depression in children and adolescents. *Int J Methods Psychiatr Res, 5*, 1-12.

Eyre, O., Jones, R. B., Agha, S. S., Wootton, R. E., Thapar, A. K., Stergiakouli, E., . . . Riglin, L. (2021). Validation of the short Mood and Feelings Questionnaire in young adulthood. *medRxiv*, 2021.2001.2022.21250311. doi:10.1101/2021.01.22.21250311

Goodman, R. (1997). The Strengths and Difficulties Questionnaire: A Research Note. *Journal of Child Psychology and Psychiatry, 38*(5), 581-586. doi:<https://doi.org/10.1111/j.1469-7610.1997.tb01545.x>

Goodman, R. (2001). Psychometric properties of the strengths and difficulties questionnaire. *J Am Acad Child Adolesc Psychiatry, 40*(11), 1337-1345. doi:10.1097/00004583-200111000-00015

Riglin, L., Agha, S. S., Eyre, O., Jones, R. B., Wootton, R. E., Thapar, A. K., . . . Thapar, A. (2021). Investigating the validity of the Strengths and Difficulties Questionnaire to assess ADHD in young adulthood. *medRxiv*, 2021.2002.2002.20248239. doi:10.1101/2021.02.02.20248239

Turner, N., Joinson, C., Peters, T. J., Wiles, N., & Lewis, G. (2014). Validity of the Short Mood and Feelings Questionnaire in late adolescence. *Psychol Assess, 26*(3), 752-762. doi:10.1037/a0036572

**Table S1.** Table summary of ages of ALSPAC participants at completion of questionnaires. Note IQR = Interquartile Range

|  | Total |  |  |  | August |  |  |  | September | |  |  |
| --- | --- | --- | --- | --- | --- | --- | --- | --- | --- | --- | --- | --- |
| SDQ Total Difficulties | N | Mean Age (Months) | SD | IQR | N | Mean Age (Months) | SD | IQR | N | Mean Age (Months) | SD | IQR |
| Timepoint (years) | |  |  |  |  |  |  |  |  |  |  |  |
| 4 | 9243 | 47.95 | 1.47 | 47-48 | 883 | 47.83 | 1.33 | 47-48 | 926 | 47.77 | 1.47 | 47-48 |
| 7 | 8296 | 81.45 | 1.36 | 81-82 | 784 | 81.27 | 1.45 | 81-81 | 819 | 81.30 | 1.11 | 81-81 |
| 8 | 7881 | 98.42 | 3.08 | 97-99 | 720 | 98.21 | 2.71 | 97-98 | 750 | 97.98 | 2.45 | 97-98 |
| 9 | 8109 | 115.81 | 1.57 | 115-116 | 755 | 115.59 | 1.50 | 115-116 | 771 | 115.57 | 1.37 | 115-116 |
| 11 | 7374 | 140.62 | 1.64 | 140-141 | 659 | 140.46 | 1.27 | 140-140 | 690 | 140.51 | 1.49 | 140-141 |
| 13 | 7059 | 157.92 | 2.19 | 157-158 | 621 | 157.95 | 2.35 | 157-158 | 664 | 157.80 | 1.95 | 157-158 |
| 16 | 5644 | 202.06 | 4.33 | 198-205 | 530 | 202.46 | 4.42 | 198-206 | 539 | 202.23 | 3.93 | 198-205 |
| 25 | 4681 | 316.79 | 6.11 | 312-322 | 413 | 317.08 | 6.24 | 311-323 | 458 | 316.04 | 6.19 | 310-322 |
| Self SMFQ | N | Mean Age (Months) | SD | IQR | N | Mean Age (Months) | SD | IQR | N | Mean Age (Months) | SD | IQR |
| Timepoint (years) | |  |  |  |  |  |  |  |  |  |  |  |
| 10 | 7430 | 127.77 | 3.17 | 126-129 | 655 | 127.85 | 3.25 | 126-129 | 732 | 127.85 | 3.05 | 126-129 |
| 13 | 6720 | 153.73 | 2.77 | 152-155 | 611 | 152.97 | 2.59 | 151-154 | 653 | 153.30 | 2.84 | 151-155 |
| 14 | 6042 | 166.02 | 2.50 | 165-167 | 554 | 166.40 | 2.39 | 165-167 | 608 | 166.12 | 2.27 | 165-167 |
| 16 | 5064 | 200.15 | 2.84 | 198-202 | 458 | 201.86 | 4.18 | 198-206 | 481 | 201.73 | 3.73 | 198-205 |
| 17 | 4446 | 214.07 | 4.72 | 211-216 | 390 | 214.28 | 4.62 | 212-216 | 463 | 214.42 | 4.59 | 212-216 |
| 18 | 3335 | 223.81 | 5.90 | 219-229 | 301 | 224.06 | 5.98 | 218-229 | 323 | 222.79 | 5.75 | 217-228 |
| 21 | 3413 | 263.41 | 6.27 | 258-269 | 320 | 263.32 | 6.20 | 257-269 | 338 | 262.27 | 6.24 | 256-268 |
| 22 | 3969 | 274.86 | 6.37 | 270-280 | 366 | 274.70 | 6.27 | 268-280 | 380 | 273.61 | 6.34 | 267-279 |
| 23 | 4025 | 286.50 | 6.23 | 281-292 | 350 | 286.44 | 6.27 | 280-292 | 390 | 285.11 | 6.13 | 279-291 |
| 25 | 4,329 | 309.17 | 6.12 | 304-314 | 384 | 309.15 | 6.05 | 303-315 | 413 | 307.95 | 6.04 | 302-314 |

**Appendix S2.** Multiple imputation

Multiple imputation by chained equations was used to impute missing SDQ and SMFQ data (continuous scores) at each time-point. We included auxiliary variables that predicted missingness (Table S1) together with variables in the analysis model (Parent-rated SDQ total difficulties (4-25 years, self-rated SMFQ (10-25 years), parent-rated SMFQ (9-16 years), maternal depression, Maternal age, gestation, birthweight, birth size, alcohol use in last 2 months of pregnancy, smoking, caesarean status, crowding, home ownership status, mother’s education, parity, and age at completion of questionnaire). We assessed Monte Carlo errors after 200 imputations and found that Monte Carlo error estimates conformed to the guidelines set by White, Royston & Wood (2011)

We conducted separate analyses by outcome variable (parent-report SDQ, self-report SMFQ, parent-report SMFQ). Variables in the analysis model were:

Maternal depression, maternal age, gestation, birthweight, birth size, alcohol use in last 2 months of pregnancy, smoking, caesarean status, crowding, home ownership status, mother’s education, week of birth within the year, age at completion of questionnaire, outcome variable (parent-report SDQ, self-report SMFQ, parent-report SMFQ)

References:

White, I. R., Royston, P., & Wood, A. M. (2011). Multiple imputation using chained equations: Issues and guidance for practice. *Statistics in Medicine, 30*(4), 377-399. doi:<https://doi.org/10.1002/sim.4067>

**Table S2.** Multiple Imputation variables, models used, and percentage of data missing from these variables/models

| Variable | Model Used | % Missing |
| --- | --- | --- |
| SDQ Maternal Depression (EPDS) | Linear Regression | 13.6 |
| SDQ Age of Mother at Birth | Linear Regression | 10.1 |
| SDQ Gestation | Linear Regression | 3.8 |
| SDQ Birthweight | Linear Regression | 4.9 |
| SDQ Birth Size | Linear Regression | 0.01 |
| SDQ Alcohol Use in Last 2 Months of Pregnancy | Logistic | 10.4 |
| SDQ Caesarean | Logistic | 10.4 |
| SDQ Crowding | Logistic | 8.4 |
| SDQ Home Ownership | Logistic | 7.1 |
| SDQ Mother’s Education | Multinomial logistic | 12.2 |
| SDQ Parity | Logistic | 7.6 |
| SDQ Smoking During Last 2 Months of Pregnancy | Logistic | 10.0 |
| SDQ Total Difficulties – 4 Years | Linear Regression. Covariates used: , maternal depression, Maternal age (years), gestation (weeks), birthweight (g), birth size (singleton/multiple), alcohol use in last 2 months of pregnancy (y/n), smoking in last 2 months of pregnancy (y/n), caesarean status (y/n), crowding, home ownership status (owned/not owned), mother’s education (degree/no degree), parity, age at completion of questionnaire (months) | 16.2 |
| SDQ Total Difficulties – 7 Years | Linear Regression Covariates used: , maternal depression, Maternal age (years), gestation (weeks), birthweight (g), birth size (singleton/multiple), alcohol use in last 2 months of pregnancy (y/n), smoking in last 2 months of pregnancy (y/n), caesarean status (y/n), crowding, home ownership status (owned/not owned), mother’s education (degree/no degree), parity, age at completion of questionnaire (months) | 25.5 |
| SDQ Total Difficulties – 8 Years | Linear Regression Covariates used: , maternal depression, Maternal age (years), gestation (weeks), birthweight (g), birth size (singleton/multiple), alcohol use in last 2 months of pregnancy (y/n), smoking in last 2 months of pregnancy (y/n), caesarean status (y/n), crowding, home ownership status (owned/not owned), mother’s education (degree/no degree), parity, age at completion of questionnaire (months) | 31.0 |
| SDQ Total Difficulties – 9 Years | Linear Regression Covariates used: , maternal depression, Maternal age (years), gestation (weeks), birthweight (g), birth size (singleton/multiple), alcohol use in last 2 months of pregnancy (y/n), smoking in last 2 months of pregnancy (y/n), caesarean status (y/n), crowding, home ownership status (owned/not owned), mother’s education (degree/no degree), parity, age at completion of questionnaire (months) | 28.6 |
| SDQ Total Difficulties – 11 Years | Linear Regression Covariates used: , maternal depression, Maternal age (years), gestation (weeks), birthweight (g), birth size (singleton/multiple), alcohol use in last 2 months of pregnancy (y/n), smoking in last 2 months of pregnancy (y/n), caesarean status (y/n), crowding, home ownership status (owned/not owned), mother’s education (degree/no degree), parity, age at completion of questionnaire (months) | 34.8 |
| SDQ Total Difficulties – 13 Years | Linear Regression Covariates used: , maternal depression, Maternal age (years), gestation (weeks), birthweight (g), birth size (singleton/multiple), alcohol use in last 2 months of pregnancy (y/n), smoking in last 2 months of pregnancy (y/n), caesarean status (y/n), crowding, home ownership status (owned/not owned), mother’s education (degree/no degree), parity, age at completion of questionnaire (months) | 37.6 |
| SDQ Total Difficulties – 16 Years | Linear Regression Covariates used: , maternal depression, Maternal age (years), gestation (weeks), birthweight (g), birth size (singleton/multiple), alcohol use in last 2 months of pregnancy (y/n), smoking in last 2 months of pregnancy (y/n), caesarean status (y/n), crowding, home ownership status (owned/not owned), mother’s education (degree/no degree), parity, age at completion of questionnaire (months) | 50.0 |
| SDQ Total Difficulties –25 Years | Linear Regression Covariates used: , maternal depression, Maternal age (years), gestation (weeks), birthweight (g), birth size (singleton/multiple), alcohol use in last 2 months of pregnancy (y/n), smoking in last 2 months of pregnancy (y/n), caesarean status (y/n), crowding, home ownership status (owned/not owned), mother’s education (degree/no degree), parity, age at completion of questionnaire (months) | 63.3 |
| Variable | Model Used | % Missing |
| Self-Rated SMFQ- Maternal Depression (EPDS) | Linear Regression | 16.2 |
| Self-Rated SMFQ- Age of Mother at Birth | Linear Regression | 13.1 |
| Self-Rated SMFQ- Gestation | Linear Regression | 6.8 |
| Self-Rated SMFQ- Birthweight | Linear Regression | 7.9 |
| Self-Rated SMFQ- Birth Size | Linear Regression | 0.2 |
| Self-Rated SMFQ- Alcohol Use in Last 2 Months of Pregnancy | Logistic | 13.5 |
| Self-Rated SMFQ- Caesarean | Logistic | 13.5 |
| Self-Rated SMFQ- Crowding | Logistic | 11.1 |
| Self-Rated SMFQ- Home Ownership | Logistic | 10.1 |
| Self-Rated SMFQ- Mother’s Education | Multinomial logistic | 14.8 |
| Self-Rated SMFQ- Parity | Logistic | 10.4 |
| SDQ Smoking During Last 2 Months of Pregnancy | Logistic | 13.1 |
| Self-Rated SMFQ – 10 Years | Linear Regression Covariates used: , maternal depression, Maternal age (years), gestation (weeks), birthweight (g), birth size (singleton/multiple), alcohol use in last 2 months of pregnancy (y/n), smoking in last 2 months of pregnancy (y/n), caesarean status (y/n), crowding, home ownership status (owned/not owned), mother’s education (degree/no degree), parity, age at completion of questionnaire (months) | 23.5 |
| Self-Rated SMFQ – 13 Years | Linear Regression Covariates used: , maternal depression, Maternal age (years), gestation (weeks), birthweight (g), birth size (singleton/multiple), alcohol use in last 2 months of pregnancy (y/n), smoking in last 2 months of pregnancy (y/n), caesarean status (y/n), crowding, home ownership status (owned/not owned), mother’s education (degree/no degree), parity, age at completion of questionnaire (months) | 30.2 |
| Self-Rated SMFQ – 14 Years | Linear Regression Covariates used: , maternal depression, Maternal age (years), gestation (weeks), birthweight (g), birth size (singleton/multiple), alcohol use in last 2 months of pregnancy (y/n), smoking in last 2 months of pregnancy (y/n), caesarean status (y/n), crowding, home ownership status (owned/not owned), mother’s education (degree/no degree), parity, age at completion of questionnaire (months) | 37.4 |
| Self-Rated SMFQ – 16 Years | Linear Regression Covariates used: , maternal depression, Maternal age (years), gestation (weeks), birthweight (g), birth size (singleton/multiple), alcohol use in last 2 months of pregnancy (y/n), smoking in last 2 months of pregnancy (y/n), caesarean status (y/n), crowding, home ownership status (owned/not owned), mother’s education (degree/no degree), parity, age at completion of questionnaire (months) | 47.8 |
| Self-Rated SMFQ – 17 Years | Linear Regression Covariates used: , maternal depression, Maternal age (years), gestation (weeks), birthweight (g), birth size (singleton/multiple), alcohol use in last 2 months of pregnancy (y/n), smoking in last 2 months of pregnancy (y/n), caesarean status (y/n), crowding, home ownership status (owned/not owned), mother’s education (degree/no degree), parity, age at completion of questionnaire (months) | 65.2 |
| Self-Rated SMFQ – 18 Years | Linear Regression Covariates used: , maternal depression, Maternal age (years), gestation (weeks), birthweight (g), birth size (singleton/multiple), alcohol use in last 2 months of pregnancy (y/n), smoking in last 2 months of pregnancy (y/n), caesarean status (y/n), crowding, home ownership status (owned/not owned), mother’s education (degree/no degree), parity, age at completion of questionnaire (months) | 53.1 |
| Self-Rated SMFQ – 21 Years | Linear Regression Covariates used: , maternal depression, Maternal age (years), gestation (weeks), birthweight (g), birth size (singleton/multiple), alcohol use in last 2 months of pregnancy (y/n), smoking in last 2 months of pregnancy (y/n), caesarean status (y/n), crowding, home ownership status (owned/not owned), mother’s education (degree/no degree), parity, age at completion of questionnaire (months) | 65.5 |
| Self-Rated SMFQ – 22 Years | Linear Regression Covariates used: , maternal depression, Maternal age (years), gestation (weeks), birthweight (g), birth size (singleton/multiple), alcohol use in last 2 months of pregnancy (y/n), smoking in last 2 months of pregnancy (y/n), caesarean status (y/n), crowding, home ownership status (owned/not owned), mother’s education (degree/no degree), parity, age at completion of questionnaire (months) | 59.1 |
| Self-Rated SMFQ – 23 Years | Linear Regression Covariates used: , maternal depression, Maternal age (years), gestation (weeks), birthweight (g), birth size (singleton/multiple), alcohol use in last 2 months of pregnancy (y/n), smoking in last 2 months of pregnancy (y/n), caesarean status (y/n), crowding, home ownership status (owned/not owned), mother’s education (degree/no degree), parity, age at completion of questionnaire (months) | 58.1 |
| Self-Rated SMFQ – 25 Years | Linear Regression Covariates used: , maternal depression, Maternal age (years), gestation (weeks), birthweight (g), birth size (singleton/multiple), alcohol use in last 2 months of pregnancy (y/n), smoking in last 2 months of pregnancy (y/n), caesarean status (y/n), crowding, home ownership status (owned/not owned), mother’s education (degree/no degree), parity, age at completion of questionnaire (months) | 58.2 |
| Parent-Rated SMFQ- Maternal Depression (EPDS) | Linear Regression | 13.4 |
| Parent -Rated SMFQ- Age of Mother at Birth | Linear Regression | 9.6 |
| Parent -Rated SMFQ- Gestation | Linear Regression | 4.4 |
| Parent -Rated SMFQ- Birthweight | Linear Regression | 5.6 |
| Parent -Rated SMFQ- Birth Size | Linear Regression | 0 |
| Parent -Rated SMFQ- Alcohol Use in Last 2 Months of Pregnancy | Logistic | 9.9 |
| Parent -Rated SMFQ- Caesarean | Logistic | 9.9 |
| Parent -Rated SMFQ- Crowding | Logistic | 8.4 |
| Parent -Rated SMFQ- Home Ownership | Logistic | 7.3 |
| Parent -Rated SMFQ- Mother’s Education | Multinomial logistic | 11.4 |
| Parent -Rated SMFQ- Parity | Logistic | 7.7 |
| Parent Smoking During Last 2 Months of Pregnancy | Logistic | 9.5 |
| Parent-Rated SMFQ – 9 Years | Linear Regression Covariates used: , maternal depression, Maternal age (years), gestation (weeks), birthweight (g), birth size (singleton/multiple), alcohol use in last 2 months of pregnancy (y/n), smoking in last 2 months of pregnancy (y/n), caesarean status (y/n), crowding, home ownership status (owned/not owned), mother’s education (degree/no degree), parity, age at completion of questionnaire (months) | 13.1 |
| Parent-Rated SMFQ – 11 Years | Linear Regression Covariates used: , maternal depression, Maternal age (years), gestation (weeks), birthweight (g), birth size (singleton/multiple), alcohol use in last 2 months of pregnancy (y/n), smoking in last 2 months of pregnancy (y/n), caesarean status (y/n), crowding, home ownership status (owned/not owned), mother’s education (degree/no degree), parity, age at completion of questionnaire (months) | 21.4 |
| Parent-Rated SMFQ – 13 Years | Linear Regression Covariates used: , maternal depression, Maternal age (years), gestation (weeks), birthweight (g), birth size (singleton/multiple), alcohol use in last 2 months of pregnancy (y/n), smoking in last 2 months of pregnancy (y/n), caesarean status (y/n), crowding, home ownership status (owned/not owned), mother’s education (degree/no degree), parity, age at completion of questionnaire (months) | 24.7 |
| Parent-Rated SMFQ – 16 Years | Linear Regression Covariates used: , maternal depression, Maternal age (years), gestation (weeks), birthweight (g), birth size (singleton/multiple), alcohol use in last 2 months of pregnancy (y/n), smoking in last 2 months of pregnancy (y/n), caesarean status (y/n), crowding, home ownership status (owned/not owned), mother’s education (degree/no degree), parity, age at completion of questionnaire (months) | 41.3 |

**Table S3.** Demographic Characteristics of the study sample

| Variable | Total |  | August |  |  | September | Aug vs Sep |
| --- | --- | --- | --- | --- | --- | --- | --- |
|  | N | Percent/Mean (SD) | N | Percent/Mean (SD) | N | Percent/Mean (SD) | Mean Diff (95%CI) |
| Age of mother at birth  (years) | 11637 | 28.34 (4.83) | 1099 | 28.49 (5.11) | 1162 | 28.13 (4.88) | .36 [-0.05, 0.77] |
| Alcohol during pregnancy  (% yes) | 11579 | 50.52% | 1092 | 49.18% | 1155 | 50.13% | -0.95 [-5.09, 3.19] |
| Birth size (% multiple) | 14625 | 1.29% | 1395 | 0.92% | 1429 | 1.24% | -0.32 [-1.08, 0.44] |
| Birthweight (grams) | 13577 | 3402.80 (549.94) | 1337 | 3403.64 (551.78) | 1358 | 3424.26 (527.87) | -20.62 [-61.40, 20.16] |
| Caesarean (% yes) | 11572 | 10.33% | 963 | 11.81% | 1040 | 9.80% | 2.01 [-0.56, 4.58] |
| Crowding (%>1) | 11737 | 6.88% | 1143 | 7.00% | 1150 | 6.81% | 0.19 [-1.81, 2.19] |
| Ethnic background  (% white) | 11286 | 94.95% | 1094 | 94.72% | 1109 | 94.79% | -0.07 [-1.74, .1.88] |
| Gestation (weeks) | 13751 | 39.43 (1.88) | 1344 | 39.48 (1.91) | 1376 | 39.52 (1.78) | -0.03 [-0.17, 0.10] |
| Home ownership  (% owned) | 12824 | 73.32% | 1256 | 73.09% | 1262 | 72.90% | -0.18 [-3.28, 3.66] |
| Maternal depression score  18 weeks (epds) | 11778 | 6.97 (4.85) | 1168 | 6.74 (4.78) | 1155 | 6.91 (4.94) | -0.18 [-0.57, 0.22] |
| Mother’s education  (% degree) | 11463 | 13.72% | 1112 | 12.77% | 1123 | 13.00% | -0.23 [-3.01, 2.55] |
| Parity (% >1) | 12721 | 55.21% | 1238 | 52.58% | 1261 | 55.11% | -2.53 [-6.44, 1.38] |
| Sex (% female) | 14643 | 48.97% | 1408 | 49.08% | 1447 | 47.20% | 1.88 [-1.79, 5.54] |
| Smoking during pregnancy  (% yes) | 11657 | 19.57% | 1099 | 20.38% | 1162 | 21.00% | -0.62[-3.96, 2.73] |

**Table S4.** Demographic information for participants born 4 (“4 weeks”) and 8 (“8 weeks”) weeks either side of the September 1^st^ Cut-off.

|  | 4 Weeks |  | 8 Weeks |  |
| --- | --- | --- | --- | --- |
|  | **N** | **Percent/Mean (SD)** | **N** | **Percent/Mean** |
| Age of Mother at Birth (Years) | 2141 | 28.30 (4.99) | 4221 | 28.43 (4.90) |
| Alcohol During Pregnancy (% Yes) | 2130 | 49.71 | 4204 | 49.19 |
| Birth Size (% Multiple) | 2698 | 1.04 | 5291 | 1.06 |
| Birthweight (grams) | 2546 | 3415.71 (540.57) | 5003 | 3407.79 (550.86) |
| Caesarean (% Yes) | 2126 | 11.10 | 4198 | 10.83 |
| Crowding (%>1) | 2332 | 6.78 | 4583 | 6.98 |
| Ethnic Background (% White) | 2201 | 94.60 | 4345 | 94.60 |
| Gestation | 2568 | 39.50 | 5046 | 39.46 (1.90) |
| Home Ownership (% Owned) | 2378 | 73.25 (1.85) | 4673 | 73.65 |
| Maternal Depression 18 weeks (EPDS) | 2197 | 6.80 (4.87) | 4300 | 6.87 (4.82) |
| Mother’s Education (% Degree) | 2112 | 13.07 | 4186 | 12.82 |
| Parity (% >1) | 2357 | 53.54 | 4632 | 55.29 |
| Sex (% Female) | 2703 | 47.80 | 5297 | 48.67 |
| Smoking During Pregnancy (% Yes) | 2141 | 20.22 | 4228 | 20.57 |

**Figure S1.** Histogram of birthdates by month. Red lines denote a period of one complete school year (September 1991-August 1992)

**Table** **S5.** Missing table patterns of SDQ total difficulties. All = No bandwidth applied, all participants considered. 4 Week = Restricted to participants born 4 weeks either side of the cut off. 8 Week = Restricted to participants born 8 weeks either side of the cut off. Limited to missingness patterns with > 1% in frequency across the sample. 1 = complete record, 0 = incomplete.

| All | Age (Years) | | | | | | | | 4 week | Age (Years) | | | | | | | | 8 week | Age (Years) | | | | | | | |
| --- | --- | --- | --- | --- | --- | --- | --- | --- | --- | --- | --- | --- | --- | --- | --- | --- | --- | --- | --- | --- | --- | --- | --- | --- | --- | --- |
| Percent | **4** | **7** | **8** | **9** | **11** | **13** | **16** | **25** | **Percent** | **4** | **7** | **8** | **9** | **11** | **13** | **16** | **25** | **Percent** | **4** | **7** | **8** | **9** | **11** | **13** | **16** | **25** |
| 17% | 1 | 1 | 1 | 1 | 1 | 1 | 1 | 1 | **16%** | 1 | 1 | 1 | 1 | 1 | 1 | 1 | 1 | **17%** | 1 | 1 | 1 | 1 | 1 | 1 | 1 | 1 |
| 24% | 0 | 0 | 0 | 0 | 0 | 0 | 0 | 0 | **25%** | 0 | 0 | 0 | 0 | 0 | 0 | 0 | 0 | **24%** | 0 | 0 | 0 | 0 | 0 | 0 | 0 | 0 |
| 9% | 1 | 1 | 1 | 1 | 1 | 1 | 1 | 0 | **8%** | 1 | 1 | 1 | 1 | 1 | 1 | 1 | 0 | **8%** | 1 | 1 | 1 | 1 | 1 | 1 | 1 | 0 |
| 6% | 1 | 0 | 0 | 0 | 0 | 0 | 0 | 0 | **6%** | 1 | 0 | 0 | 0 | 0 | 0 | 0 | 0 | **6%** | 1 | 0 | 0 | 0 | 0 | 0 | 0 | 0 |
| 4% | 1 | 1 | 1 | 1 | 1 | 1 | 0 | 0 | **4%** | 1 | 1 | 1 | 1 | 1 | 1 | 0 | 0 | **4%** | 1 | 1 | 1 | 1 | 1 | 1 | 0 | 0 |
| 3% | 1 | 1 | 0 | 0 | 0 | 0 | 0 | 0 | **3%** | 1 | 1 | 0 | 0 | 0 | 0 | 0 | 0 | **3%** | 1 | 1 | 0 | 0 | 0 | 0 | 0 | 0 |
| 2% | 1 | 1 | 1 | 1 | 0 | 0 | 0 | 0 | **2%** | 1 | 1 | 1 | 1 | 1 | 0 | 0 | 0 | **2%** | 1 | 1 | 1 | 1 | 0 | 0 | 0 | 0 |
| 2% | 1 | 1 | 1 | 1 | 1 | 1 | 0 | 1 | **2%** | 1 | 1 | 1 | 1 | 1 | 1 | 0 | 1 | **2%** | 1 | 1 | 1 | 1 | 1 | 0 | 0 | 0 |
| 2% | 1 | 1 | 1 | 1 | 1 | 0 | 0 | 0 | **2%** | 1 | 1 | 1 | 1 | 0 | 0 | 0 | 0 | **2%** | 1 | 1 | 1 | 1 | 1 | 1 | 0 | 1 |
| 2% | 1 | 1 | 0 | 1 | 0 | 0 | 0 | 0 | **2%** | 1 | 1 | 0 | 1 | 0 | 0 | 0 | 0 | **1%** | 1 | 1 | 0 | 1 | 0 | 0 | 0 | 0 |
| 1% | 0 | 1 | 0 | 0 | 0 | 0 | 0 | 0 | **1%** | 1 | 1 | 1 | 0 | 0 | 0 | 0 | 0 | **1%** | 0 | 1 | 0 | 0 | 0 | 0 | 0 | 0 |
|  |  |  |  |  |  |  |  |  | **1%** | 0 | 1 | 0 | 0 | 0 | 0 | 0 | 0 | **1%** | 1 | 1 | 1 | 0 | 0 | 0 | 0 | 0 |

**Table S6.** Missing table patterns of SDQ total difficulties. August vs September. Limited to missingness patterns with > 1% in frequency across the sample. 1 = complete record, 0 = incomplete.

| August | Age (Years) | | | | | | | | September | Age (Years) | | | | | | | |
| --- | --- | --- | --- | --- | --- | --- | --- | --- | --- | --- | --- | --- | --- | --- | --- | --- | --- |
| Percent | **4** | **7** | **8** | **9** | **11** | **13** | **16** | **25** | **Percent** | **4** | **7** | **8** | **9** | **11** | **13** | **16** | **25** |
| 16% | 1 | 1 | 1 | 1 | 1 | 1 | 1 | 1 | **16%** | 1 | 1 | 1 | 1 | 1 | 1 | 1 | 1 |
| 25% | 0 | 0 | 0 | 0 | 0 | 0 | 0 | 0 | **24%** | 0 | 0 | 0 | 0 | 0 | 0 | 0 | 0 |
| 8% | 1 | 1 | 1 | 1 | 1 | 1 | 1 | 0 | **8%** | 1 | 1 | 1 | 1 | 1 | 1 | 1 | 0 |
| 7% | 1 | 0 | 0 | 0 | 0 | 0 | 0 | 0 | **6%** | 1 | 0 | 0 | 0 | 0 | 0 | 0 | 0 |
| 4% | 1 | 1 | 1 | 1 | 1 | 1 | 0 | 0 | **4%** | 1 | 1 | 1 | 1 | 1 | 1 | 0 | 0 |
| 3% | 1 | 1 | 0 | 0 | 0 | 0 | 0 | 0 | **4%** | 1 | 1 | 0 | 0 | 0 | 0 | 0 | 0 |
| 2% | 1 | 1 | 1 | 1 | 0 | 0 | 0 | 0 | **2%** | 1 | 1 | 1 | 1 | 1 | 1 | 0 | 1 |
| 2% | 1 | 1 | 1 | 1 | 1 | 0 | 0 | 0 | **2%** | 1 | 1 | 0 | 1 | 0 | 0 | 0 | 0 |
| 2% | 1 | 1 | 1 | 1 | 1 | 1 | 0 | 1 | **2%** | 1 | 1 | 1 | 1 | 0 | 0 | 0 | 0 |
| 1% | 1 | 1 | 0 | 1 | 0 | 0 | 0 | 0 | **2%** | 1 | 1 | 1 | 1 | 1 | 0 | 0 | 0 |
| 1% | 0 | 1 | 0 | 0 | 0 | 0 | 0 | 0 | **1%** | 1 | 1 | 1 | 0 | 0 | 0 | 0 | 0 |
| 1% | 1 | 1 | 1 | 0 | 0 | 0 | 0 | 0 | **1%** | 1 | 1 | 1 | 0 | 1 | 1 | 1 | 1 |

**Table S7.** Missing table patterns of Self-rated SMFQ. All = No bandwidth applied, all participants considered. 4 Week = Restricted to participants born 4 weeks either side of the cut off. 8 Week = Restricted to participants born 8 weeks either side of the cut off. Limited to missingness patterns with > 1% in frequency across the sample. 1 = complete record, 0 = incomplete.

| **All** | Age (Years) | | | | | | | | | | **4 Week** | Age (Years) | | | | | | | | | | **8 Week** | Age (Years) | | | | | | | | | |
| --- | --- | --- | --- | --- | --- | --- | --- | --- | --- | --- | --- | --- | --- | --- | --- | --- | --- | --- | --- | --- | --- | --- | --- | --- | --- | --- | --- | --- | --- | --- | --- | --- |
| **Percent** | **10** | **13** | **14** | **16** | **17** | **18** | **21** | **22** | **23** | **25** | **Percent** | **10** | **13** | **14** | **16** | **17** | **18** | **21** | **22** | **23** | **25** | **Percent** | **10** | **13** | **14** | **16** | **17** | **18** | **21** | **22** | **23** | **25** |
| **6%** | 1 | 1 | 1 | 1 | 1 | 1 | 1 | 1 | 1 | 1 | **6%** | 1 | 1 | 1 | 1 | 1 | 1 | 1 | 1 | 1 | 1 | **6%** | 1 | 1 | 1 | 1 | 1 | 1 | 1 | 1 | 1 | 1 |
| **35%** | 0 | 0 | 0 | 0 | 0 | 0 | 0 | 0 | 0 | 0 | **37%** | 0 | 0 | 0 | 0 | 0 | 0 | 0 | 0 | 0 | 0 | **36%** | 0 | 0 | 0 | 0 | 0 | 0 | 0 | 0 | 0 | 0 |
| **5%** | 1 | 1 | 1 | 0 | 0 | 0 | 0 | 0 | 0 | 0 | **5%** | 1 | 1 | 1 | 0 | 0 | 0 | 0 | 0 | 0 | 0 | **5%** | 1 | 1 | 1 | 0 | 0 | 0 | 0 | 0 | 0 | 0 |
| **4%** | 1 | 0 | 0 | 0 | 0 | 0 | 0 | 0 | 0 | 0 | **3%** | 1 | 0 | 0 | 0 | 0 | 0 | 0 | 0 | 0 | 0 | **4%** | 1 | 0 | 0 | 0 | 0 | 0 | 0 | 0 | 0 | 0 |
| **2%** | 1 | 1 | 0 | 0 | 0 | 0 | 0 | 0 | 0 | 0 | **2%** | 1 | 1 | 0 | 0 | 0 | 0 | 0 | 0 | 0 | 0 | **2%** | 1 | 1 | 0 | 0 | 0 | 0 | 0 | 0 | 0 | 0 |
| **2%** | 1 | 1 | 1 | 0 | 1 | 0 | 0 | 0 | 0 | 0 | **2%** | 1 | 1 | 1 | 0 | 1 | 0 | 0 | 0 | 0 | 0 | **2%** | 1 | 1 | 1 | 0 | 1 | 0 | 0 | 0 | 0 | 0 |
| **2%** | 1 | 1 | 1 | 1 | 1 | 0 | 0 | 0 | 0 | 0 | **2%** | 1 | 1 | 1 | 1 | 0 | 0 | 0 | 0 | 0 | 0 | **2%** | 1 | 1 | 1 | 1 | 1 | 0 | 0 | 0 | 0 | 0 |
| **2%** | 1 | 1 | 1 | 1 | 0 | 0 | 0 | 0 | 0 | 0 | **2%** | 1 | 1 | 1 | 1 | 1 | 0 | 0 | 0 | 0 | 0 | **1%** | 1 | 1 | 1 | 1 | 0 | 0 | 0 | 0 | 0 | 0 |
| **1%** | 0 | 0 | 0 | 1 | 0 | 0 | 0 | 0 | 0 | 0 | **1%** | 1 | 1 | 1 | 1 | 1 | 1 | 1 | 1 | 1 | 0 | **1%** | 0 | 0 | 0 | 1 | 0 | 0 | 0 | 0 | 0 | 0 |
| **1%** | 1 | 1 | 1 | 1 | 1 | 1 | 1 | 1 | 0 | 1 |  |  |  |  |  |  |  |  |  |  |  | **1%** | 1 | 1 | 1 | 1 | 1 | 1 | 1 | 1 | 1 | 0 |
| **1%** | 0 | 0 | 0 | 0 | 1 | 0 | 0 | 0 | 0 | 0 |  |  |  |  |  |  |  |  |  |  |  | **1%** | 1 | 1 | 1 | 1 | 1 | 1 | 0 | 1 | 1 | 1 |

**Table S8.** Missing table patterns of Self-rated SMFQ. August = Children born in August; September = Children born in September. Limited to missingness patterns with > 1% in frequency across the sample. 1 = complete record, 0 = incomplete.

| August | Age (Years) | | | | | | | | | | September | Age (Years) | | | | | | | | | |
| --- | --- | --- | --- | --- | --- | --- | --- | --- | --- | --- | --- | --- | --- | --- | --- | --- | --- | --- | --- | --- | --- |
| Percent | **10** | **13** | **14** | **16** | **17** | **18** | **21** | **22** | **23** | **25** | **Percent** | **10** | **13** | **14** | **16** | **17** | **18** | **21** | **22** | **23** | **25** |
| 6% | 1 | 1 | 1 | 1 | 1 | 1 | 1 | 1 | 1 | 1 | **6%** | 1 | 1 | 1 | 1 | 1 | 1 | 1 | 1 | 1 | 1 |
| 40 | 0 | 0 | 0 | 0 | 0 | 0 | 0 | 0 | 0 | 0 | **35** | 0 | 0 | 0 | 0 | 0 | 0 | 0 | 0 | 0 | 0 |
| 4 | 1 | 1 | 1 | 0 | 0 | 0 | 0 | 0 | 0 | 0 | **5** | 1 | 1 | 1 | 0 | 0 | 0 | 0 | 0 | 0 | 0 |
| 3 | 1 | 0 | 0 | 0 | 0 | 0 | 0 | 0 | 0 | 0 | **3** | 1 | 0 | 0 | 0 | 0 | 0 | 0 | 0 | 0 | 0 |
| 2 | 1 | 1 | 0 | 0 | 0 | 0 | 0 | 0 | 0 | 0 | **2** | 1 | 1 | 0 | 0 | 0 | 0 | 0 | 0 | 0 | 0 |
| 2 | 1 | 1 | 1 | 1 | 1 | 0 | 0 | 0 | 0 | 0 | **2** | 1 | 1 | 1 | 0 | 1 | 0 | 0 | 0 | 0 | 0 |
| 1 | 1 | 1 | 1 | 0 | 1 | 0 | 0 | 0 | 0 | 0 | **2** | 1 | 1 | 1 | 1 | 0 | 0 | 0 | 0 | 0 | 0 |
| 1 | 1 | 1 | 1 | 1 | 0 | 0 | 0 | 0 | 0 | 0 | **1** | 1 | 1 | 1 | 1 | 1 | 0 | 0 | 0 | 0 | 0 |
| 1 | 1 | 1 | 1 | 1 | 1 | 0 | 1 | 1 | 1 | 1 | **1** | 0 | 0 | 0 | 0 | 1 | 0 | 0 | 0 | 0 | 0 |
| 1 | 0 | 1 | 0 | 0 | 0 | 0 | 0 | 0 | 0 | 0 | **1** | 0 | 0 | 0 | 1 | 0 | 0 | 0 | 0 | 0 | 0 |
|  |  |  |  |  |  |  |  |  |  |  | **1** | 1 | 1 | 1 | 1 | 1 | 1 | 1 | 1 | 0 | 1 |

**Table S9.** Missing table patterns of Parent rated SMFQ. All participants = No bandwidth applied, all participants considered. 4 Week = Restricted to participants born 4 weeks either side of the cut off. 8 Weeks = Restricted to participants born 8 weeks either side of the cut off. Limited to missingness patterns with > 1% in frequency across the sample. 1 = complete record, 0 = incomplete.

| All Participants | Age (Years) | | | | 4 Weeks | Age (Years) | | | | 8 Weeks | Age (Years) | | | |
| --- | --- | --- | --- | --- | --- | --- | --- | --- | --- | --- | --- | --- | --- | --- |
| Percent | **9** | **11** | **13** | **16** | **Percent** | **9** | **11** | **13** | **16** | **Percent** | **9** | **11** | **13** | **16** |
| 29% | 1 | 1 | 1 | 1 | **27%** | 1 | 1 | 1 | 1 | **28%** | 1 | 1 | 1 | 1 |
| 37 | 0 | 0 | 0 | 0 | **39** | 0 | 0 | 0 | 0 | **38** | 0 | 0 | 0 | 0 |
| 9 | 1 | 1 | 1 | 0 | **8** | 1 | 1 | 1 | 0 | **9** | 1 | 1 | 1 | 0 |
| 6 | 1 | 0 | 0 | 0 | **6** | 1 | 0 | 0 | 0 | **6** | 1 | 0 | 0 | 0 |
| 4 | 1 | 1 | 0 | 0 | **4** | 1 | 1 | 0 | 0 | **4** | 1 | 1 | 0 | 0 |
| 2 | 1 | 0 | 1 | 0 | **2** | 1 | 1 | 0 | 1 | **2** | 1 | 0 | 1 | 0 |
| 2 | 1 | 1 | 0 | 1 | **2** | 1 | 0 | 1 | 0 | **2** | 1 | 1 | 0 | 1 |
| 2 | 0 | 1 | 0 | 0 | **2** | 0 | 1 | 1 | 1 | **2** | 1 | 0 | 1 | 1 |
| 2 | 1 | 0 | 1 | 1 | **2** | 0 | 1 | 0 | 0 | **2** | 0 | 1 | 0 | 0 |
| 2 | 0 | 1 | 1 | 0 | **2** | 1 | 0 | 1 | 1 | **1** | 0 | 1 | 1 | 0 |
| 2 | 0 | 0 | 1 | 0 | **1** | 0 | 1 | 1 | 0 | **1** | 0 | 1 | 1 | 1 |
| 1 | 0 | 1 | 1 | 1 | **1** | 0 | 0 | 1 | 0 | **1** | 0 | 0 | 1 | 0 |
|  |  |  |  |  | **1** | 0 | 0 | 0 | 1 |  |  |  |  |  |

**Table S10.** Missing table patterns of Parent rated SMFQ. August = Children born in August; September = Children born in September. Limited to missingness patterns with > 1% in frequency across the sample. 1 = complete record, 0 = incomplete.

| August | | Age (Years) |  |  | September | | Age (Years) |  |  |
| --- | --- | --- | --- | --- | --- | --- | --- | --- | --- |
| Percent | **9 Years** | **11 Years** | **13 Years** | **16 Years** | **Percent** | **9 Years** | **11 Years** | **13 Years** | **16 Years** |
| 27% | 1 | 1 | 1 | 1 | **27%** | 1 | 1 | 1 | 1 |
| 40 | 0 | 0 | 0 | 0 | **39** | 0 | 0 | 0 | 0 |
| 8 | 1 | 1 | 1 | 0 | **9** | 1 | 1 | 1 | 0 |
| 7 | 1 | 0 | 0 | 0 | **6** | 1 | 0 | 0 | 0 |
| 4 | 1 | 1 | 0 | 0 | **4** | 1 | 1 | 0 | 0 |
| 3 | 1 | 1 | 0 | 1 | **2** | 1 | 0 | 1 | 0 |
| 2 | 1 | 0 | 1 | 0 | **2** | 0 | 1 | 0 | 0 |
| 2 | 1 | 0 | 1 | 1 | **2** | 0 | 1 | 1 | 1 |
| 1 | 0 | 1 | 1 | 0 | **2** | 1 | 1 | 0 | 1 |
| 1 | 0 | 1 | 0 | 0 | **2** | 1 | 0 | 1 | 1 |
| 1 | 0 | 1 | 1 | 1 | **2** | 0 | 0 | 1 | 0 |
|  |  |  |  |  | **1** | 0 | 0 | 0 | 1 |
|  |  |  |  |  | **1** | 0 | 1 | 1 | 0 |
|  |  |  |  |  | **1** | 1 | 0 | 0 | 1 |

**Table S11.** Logistic regressions of covariates on being a complete-case (i.e., having all data at all mental health timepoints). Ns listed are based on participants with complete records at all timepoints in these outcomes.

|  | SDQ (N = 2190) | | | Self-rated SMFQ (N = 743) | | | Parent-rated SMFQ (N = 3444) | | |
| --- | --- | --- | --- | --- | --- | --- | --- | --- | --- |
| Variable | **Coef.** | **[95% CI]** | **p** | **Coef.** | **[95% CI]** | **p** | **Coef.** | **[95% CI]** | **p** |
| Age within school year | 0.01 | [-0.13, 0.16] | 0.86 | -0.06 | [-0.29, 0.18] | 0.65 | 0.04 | [-0.08, 0.16] | 0.52 |
| Sex (1=F) | 0.16 | [0.07, 0.25] | <0.001 | 0.85 | [0.69, 1.01] | <0.001 | 0.11 | [0.04, 0.19] | <0.01 |
| Month of Birth | 0.00 | [-0.01, 0.01] | 1.00 | 0.00 | [-0.02, 0.02] | 0.93 | 0.00 | [-0.01, 0.01] | 0.94 |
| Birth Size (Single/Multiple) | -0.88 | [-1.44, -0.31] | <0.01 | -0.33 | [-1.09, 0.42] | 0.39 | -0.71 | [-1.13, -0.29] | <0.01 |
| Mother's Education (No Degree/Degree) | 0.95 | [0.83, 1.07] | <0.001 | 0.93 | [0.76, 1.10] | <0.001 | 0.88 | [0.77, 0.99] | <0.001 |
| Home Ownership (Not Owned/Owned) | 1.34 | [1.20, 1.49] | <0.001 | 1.52 | [1.25, 1.79] | <0.001 | 1.37 | [1.25, 1.49] | <0.001 |
| Crowding Index (<1/1) | -1.59 | [-1.92, -1.26] | <0.001 | -1.26 | [-1.76, -0.76] | <0.001 | -1.46 | [-1.70, -1.22] | <0.001 |
| Parity (First/Not First-born) | -0.17 | [-0.26, -0.08] | <0.001 | -0.27 | [-0.42, -0.12] | <0.001 | -0.22 | [-0.30, -0.14] | <0.001 |
| Alcohol During Pregnancy (No/Yes) | 0.42 | [0.33, 0.52] | <0.001 | 0.29 | [0.14, 0.44] | <0.001 | 0.39 | [0.31, 0.47] | <0.001 |
| Birthweight (g) | 0.0002 | [0.0001, 0.0003] | <0.001 | 0.0000 | [-0.0001, 0.0002] | 0.82 | 0.0002 | [0.0002, 0.0003] | <0.001 |
| Gestation (wks) | 0.04 | [0.01, 0.06] | <0.01 | 0.04 | [0.00, 0.08] | 0.06 | 0.04 | [0.02, 0.06] | <0.001 |
| Smoking in Pregnancy (No/Yes) | -1.12 | [-1.28, -0.96] | <0.001 | -1.09 | [-1.36, -0.82] | <0.001 | -1.05 | [-1.18, -0.93] | <0.001 |
| Age of mother at birth | 0.09 | [0.08, 0.10] | <0.001 | 0.10 | [0.08, 0.11] | <0.001 | 0.09 | [0.08, 0.10] | <0.001 |
| Caesarean (No/Yes) | -0.09 | [-0.24, 0.07] | 0.27 | 0.08 | [-0.16, 0.32] | 0.51 | -0.05 | [-0.18, 0.08] | 0.44 |
| Ethnicity (white/non-white) | -0.73 | [-1.00, -0.46] | <0.001 | -0.53 | [-0.95, -0.11] | 0.01 | -0.81 | [-1.03, -0.58] | <0.001 |
| Maternal Depression Score (EPDS) | -0.06 | [-0.07, -0.05] | <0.001 | -0.06 | [-0.08, -0.04] | <0.001 | -0.06 | [-0.07, -0.05] | <0.001 |

**Table S12.** Descriptive statistics of outcome measures in August and September born children (Parent-rated SDQ Total Difficulties, self-rated and parent-rated SMFQ, unstandardized, complete-case data).

| Measure | Total |  | August |  | September |  |
| --- | --- | --- | --- | --- | --- | --- |
| SDQ Total Difficulties | N | M (SD) | N | M (SD) | N | M (SD) |
| 4 years | 9,312 | 8.92 (4.59) | 894 | 9.21 (4.53) | 935 | 9.28 (4.61) |
| 7 years | 8,281 | 7.48 (4.77) | 784 | 7.91 (4.97) | 817 | 7.27 (4.69) |
| 8 years | 7,669 | 7.84 (5.21) | 699 | 8.34 (5.41) | 722 | 7.61 (5.29) |
| 9 years | 7,934 | 6.88 (4.96) | 732 | 7.34 (5.24) | 754 | 6.77 (5.03) |
| 11 years | 7,253 | 6.56 (4.98) | 648 | 6.96 (5.19) | 683 | 6.28 (4.91) |
| 13 years | 6,933 | 6.81 (5.00) | 610 | 7.09 (5.22) | 658 | 6.51 (4.89) |
| 16 years | 5,554 | 6.15 (4.77) | 523 | 6.42 (4.88) | 533 | 6.10 (4.81) |
| 25 Years | 4,076 | 5.65 (5.11) | 365 | 5.04 (4.50) | 399 | 5.18 (4.64) |
| Self-Rated SMFQ |  |  |  |  |  |  |
| 10 Years | 7,245 | 4.03 (3.50) | 630 | 4.06 (3.61) | 715 | 3.99 (3.61) |
| 13 Years | 6,607 | 3.97 (3.85) | 602 | 3.76 (3.46) | 645 | 4.00 (3.91) |
| 14 Years | 5,925 | 4.92 (4.48) | 545 | 5.04 (4.51) | 597 | 4.51 (4.36) |
| 16 Years | 4,939 | 5.91 (5.64) | 452 | 6.07 (5.55) | 465 | 6.21 (5.98) |
| 17 Years | 3,299 | 6.81 (5.90) | 296 | 6.55 (5.49) | 320 | 7.02 (6.36) |
| 18 Years | 4,444 | 6.59 (5.24) | 390 | 6.72 (5.17) | 463 | 7.04 (5.59) |
| 21 Years | 3,271 | 5.30 (5.10) | 305 | 5.65 (5.30) | 318 | 5.72 (5.49) |
| 22 Years | 3,869 | 6.20 (5.53) | 354 | 6.32 (5.38) | 367 | 6.15 (5.31) |
| 23 Years | 3,972 | 7.03 (6.05) | 347 | 6.99 (5.79) | 390 | 7.17 (6.30) |
| 25 Years | 3,962 | 6.88 (6.41) | 358 | 7.16 (6.48) | 375 | 6.74 (6.34) |
| Parent-Rated SMFQ |  |  |  |  |  |  |
| 9 Years | 7,966 | 2.59 (3.26) | 737 | 2.74 (3.46) | 756 | 2.58 (3.21) |
| 11 Years | 7,201 | 2.34 (3.23) | 642 | 2.60 (3.39) | 682 | 2.33 (3.41) |
| 13 Years | 6,899 | 2.36 (3.32) | 605 | 2.52 (3.56) | 650 | 2.32 (3.28) |
| 16 Years | 5,383 | 2.14 (3.40) | 503 | 2.16 (3.38) | 517 | 2.31 (3.71) |

**Table S13.** Descriptive statistics of outcome measures (Parent-rated SDQ Total Difficulties, self-rated and parent-rated SMFQ, unstandardized, complete-case data; restricted to 4 week bandwidth (left) and 8 week bandwidth (right)).

|  | 4 weeks |  | 8 weeks |  |
| --- | --- | --- | --- | --- |
| Variable | N | Mean (SD) | N | Mean (SD) |
| SDQ Total Difficulties | |  |  |  |
| 4 Years | 1,734 | 9.25 (4.56) | 3,453 | 9.10 (4.63) |
| 7 Years | 1,527 | 7.57 (4.86) | 3,026 | 7.51 (4.80) |
| 8 Years | 1,356 | 7.95 (5.38) | 2,681 | 7.82 (5.22) |
| 9 Years | 1,417 | 7.05 (5.12) | 2,867 | 6.85 (5.06) |
| 11 Years | 1,273 | 6.63 (5.06) | 2,577 | 6.52 (4.97) |
| 13 Years | 1,210 | 6.79 (5.06) | 2,444 | 6.64 (4.92) |
| 16 Years | 1,000 | 6.25 (4.83 | 2,001 | 6.08 (4.71) |
| 25 Years | 728 | 5.13 (4.55) | 1,458 | 5.27 (4.79) |
| Self-rated SMFQ | |  |  |  |
| 10 Years | 1,282 | 4.05 (3.65) | 2,573 | 4.02 (3.48) |
| 13 Years | 1,186 | 3.90 (3.70) | 2,358 | 3.86 (3.68) |
| 14 Years | 1,087 | 4.77 (4.43) | 2,139 | 4.77 (4.41) |
| 16 Years | 867 | 6.09 (5.71) | 1,774 | 6.04 (5.77) |
| 17 Years | 585 | 6.83 (5.96) | 1,177 | 6.77 (5.99) |
| 18 Years | 805 | 6.86 (5.34) | 1,630 | 6.66 (5.29) |
| 21 Years | 589 | 5.80 (5.43) | 1,181 | 5.40 (5.23) |
| 22 Years | 685 | 6.24 (5.34) | 1,370 | 6.14 (5.46) |
| 23 Years | 694 | 7.09 (6.06) | 1,409 | 7.02 (6.06) |
| 25 Years | 692 | 6.98 (6.40) | 1,407 | 6.82 (6.29) |
| Parent-rated SMFQ | |  |  |  |
| 9 Years | 1,421 | 2.65 (3.33) | 2,882 | 2.60 (3.32) |
| 11 Years | 1,265 | 2.47 (3.44) | 2,554 | 2.40 (3.29) |
| 13 Years | 1,196 | 2.41 (3.44) | 2,433 | 2.29 (3.24) |
| 16 Years | 966 | 2.25 (3.59) | 1,928 | 2.17 (3.44) |

**Table S14.** Regression results for SDQ total difficulties and subscales by relative age, Imputed data (N=11116) The numbers contained in this table correspond to figure 2. Coefficient represents difference in standardised parent-report SDQ score between children born between 1st September-31st August. “Unadjusted Model” = Age within School Year entered in the regression alone. “All Covariates” = Model after adjustments for all covariates.

|  | Unadjusted |  |  | All Covariates |  |  |
| --- | --- | --- | --- | --- | --- | --- |
| Total | Coef. | 95% CI. | p | Coef. | 95% CI. | p |
| 4 Years | 0.02 | [-0.05, 0.08] | 0.61 | 0.03 | [-0.03, 0.09] | 0.33 |
| 7 Years | 0.15 | [0.08, 0.22] | <0.001 | 0.16 | [0.10, 0.23] | <0.001 |
| 8 Years | 0.18 | [0.11, 0.25] | <0.001 | 0.18 | [0.11, 0.24] | <0.001 |
| 9 Years | 0.17 | [0.10, 0.23] | <0.001 | 0.17 | [0.11, 0.24] | <0.001 |
| 11 Years | 0.22 | [0.15, 0.29] | <0.001 | 0.23 | [0.17, 0.30] | <0.001 |
| 13 Years | 0.14 | [0.07, 0.21] | <0.001 | 0.15 | [0.08, 0.22] | <0.001 |
| 16 Years | 0.09 | [0.01, 0.17] | 0.03 | 0.10 | [0.02, 0.18] | 0.01 |
| 25 Years | -0.01 | [-0.10, 0.09] | 0.89 | 0.01 | [-0.09, 0.11] | 0.86 |
| Conduct |  |  |  |  |  |  |
| 4 Years | <0.01 | [-0.06, 0.06] | 0.98 | 0.01 | [-0.05, 0.07] | 0.74 |
| 7 Years | -0.02 | [-0.08, 0.05] | 0.64 | -0.01 | [-0.07, 0.06] | 0.82 |
| 8 Years | 0.03 | [-0.04, 0.10] | 0.36 | 0.04 | [-0.03, 0.10] | 0.27 |
| 9 Years | -0.03 | [-0.09, 0.04] | 0.46 | -0.02 | [-0.09, 0.05] | 0.58 |
| 11 Years | 0.08 | [0.01, 0.15] | 0.03 | 0.09 | [0.02, 0.16] | 0.01 |
| 13 Years | 0.02 | [-0.05, 0.09] | 0.63 | 0.02 | [-0.05, 0.09] | 0.52 |
| 16 Years | <0.01 | [-0.08, 0.09] | 0.93 | 0.01 | [-0.07, 0.09] | 0.76 |
| 25 Years | -0.02 | [-0.11, 0.07] | 0.71 | -0.01 | [-0.10, 0.08] | 0.88 |
| Emotional |  |  |  |  |  |  |
| 4 Years | 0.09 | [0.02, 0.15] | 0.01 | 0.09 | [0.03, 0.16] | 0.01 |
| 7 Years | 0.13 | [0.06, 0.20] | <0.001 | 0.13 | [0.07, 0.20] | <0.001 |
| 8 Years | 0.09 | [0.03, 0.16] | 0.01 | 0.08 | [0.02, 0.15] | 0.01 |
| 9 Years | 0.12 | [0.05, 0.19] | <0.01 | 0.12 | [0.05, 0.19] | <0.001 |
| 11 Years | 0.11 | [0.04, 0.18] | <0.01 | 0.11 | [0.04, 0.18] | <0.01 |
| 13 Years | 0.08 | [0.01, 0.16] | 0.03 | 0.08 | [0.01, 0.16] | 0.03 |
| 16 Years | 0.09 | [0.02, 0.17] | 0.02 | 0.09 | [0.02, 0.17] | 0.02 |
| 25 Years | -0.02 | [-0.11, 0.07] | 0.70 | -0.01 | [-0.10, 0.07] | 0.74 |
| Hyperactivity |  |  |  |  |  |  |
| 4 Years | <0.01 | [-0.07, 0.06] | 0.91 | 0.01 | [-0.05, 0.07] | 0.81 |
| 7 Years | 0.18 | [0.11, 0.24] | <0.001 | 0.18 | [0.12, 0.25] | <0.001 |
| 8 Years | 0.22 | [0.16, 0.29] | <0.001 | 0.23 | [0.16, 0.29] | <0.001 |
| 9 Years | 0.19 | [0.13, 0.26] | <0.001 | 0.20 | [0.14, 0.26] | <0.001 |
| 11 Years | 0.25 | [0.18, 0.32] | <0.001 | 0.26 | [0.20, 0.33] | <0.001 |
| 13 Years | 0.17 | [0.10, 0.24] | <0.001 | 0.18 | [0.11, 0.25] | <0.001 |
| 16 Years | 0.07 | [-0.01, 0.15] | 0.08 | 0.08 | [<0.01, 0.16] | 0.04 |
| 25 Years | -0.01 | [-0.10, 0.08] | 0.82 | <0.01 | [-0.09, 0.09] | 0.95 |
| Peer |  |  |  |  |  |  |
| 4 Years | -0.03 | [-0.09, 0.04] | 0.42 | -0.02 | [-0.08, 0.05] | 0.58 |
| 7 Years | 0.08 | [0.02, 0.15] | 0.02 | 0.09 | [0.02, 0.16] | 0.01 |
| 8 Years | 0.11 | [0.04, 0.18] | <0.01 | 0.10 | [0.03, 0.17] | 0.01 |
| 9 Years | 0.15 | [0.08, 0.22] | <0.001 | 0.15 | [0.09, 0.22] | <0.001 |
| 11 Years | 0.15 | [0.08, 0.22] | <0.001 | 0.16 | [0.09, 0.23] | <0.001 |
| 13 Years | 0.12 | [0.05, 0.19] | <0.01 | 0.12 | [0.05, 0.19] | <0.01 |
| 16 Years | 0.05 | [-0.03, 0.13] | 0.25 | 0.05 | [-0.03, 0.13] | 0.18 |
| 25 Years | -0.01 | [-0.10, 0.09] | 0.87 | 0.01 | [-0.09, 0.10] | 0.91 |

**Table S15.** Regression results for Self-rated SMFQ by relative age, Imputed data (N=9468) The numbers contained in this table correspond to figure 3. Coefficient represents difference in standardised self-report SMFQ score between children born between 1st September-31st August. “Unadjusted Model” = Age within School Year entered in the regression alone. “All Covariates” = Model after adjustments for all covariates.

|  | Unadjusted |  |  | All Covariates |  |  |
| --- | --- | --- | --- | --- | --- | --- |
|  | **Coef.** | **95% CI** | **P** | **Coef.** | **95% CI** | **p** |
| 10 Years | 0.02 | [-0.06, 0.09] | 0.64 | 0.02 | [-0.05, 0.10] | 0.54 |
| 13 Years | 0.01 | [-0.07, 0.09] | 0.84 | 0.01 | [-0.07, 0.09] | 0.82 |
| 14 Years | 0.12 | [0.04, 0.20] | <0.01 | 0.12 | [0.04, 0.20] | <0.01 |
| 16 Years | 0.07 | [-0.02, 0.15] | 0.12 | 0.07 | [-0.01, 0.15] | 0.10 |
| 17 Years | -0.08 | [-0.18, 0.02] | 0.13 | -0.06 | [-0.17, 0.04] | 0.22 |
| 18 Years | -0.03 | [-0.12, 0.06] | 0.47 | -0.02 | [-0.11, 0.07] | 0.63 |
| 21 Years | 0.04 | [-0.06, 0.14] | 0.43 | 0.04 | [-0.05, 0.14] | 0.38 |
| 22 Years | 0.07 | [-0.03, 0.16] | 0.17 | 0.07 | [-0.02, 0.17] | 0.13 |
| 23 Years | 0.02 | [-0.08, 0.11] | 0.62 | 0.04 | [-0.06, 0.13] | 0.45 |
| 25 Years | 0.14 | [0.04, 0.23] | 0.01 | 0.15 | [0.05, 0.24] | <0.01 |

**Table S16.** Regression results for parent-rated SMFQ by relative age, Imputed data (N=9164) The numbers contained in this table correspond to figure 4. Coefficient represents difference in standardised parent-report SMFQ score between children born between 1st September-31st August. “Unadjusted Model” = Age within School Year entered in the regression alone. “All Covariates” = Model after adjustments for all covariates.

|  | Unadjusted |  |  | All Covariates |  |  |
| --- | --- | --- | --- | --- | --- | --- |
|  | **Coef.** | **95% CI** | **p** | **Coef.** | **95% CI** | **p** |
| 9 Years | 0.12 | [0.05, 0.19] | 0.00 | 0.12 | [0.05, 0.19] | 0.00 |
| 11 Years | 0.16 | [0.09, 0.23] | <0.001 | 0.17 | [0.09, 0.24] | <0.001 |
| 13 Years | 0.04 | [-0.04, 0.11] | 0.36 | 0.04 | [-0.04, 0.11] | 0.30 |
| 16 Years | 0.05 | [-0.03, 0.14] | 0.23 | 0.05 | [-0.03, 0.14] | 0.22 |

**Table S17.** Regression results for parent-rated SDQ total difficulties scores by relative age, restricted to 4 weeks either side of September 1^st^ cut-off, Imputed data (N=2035) The numbers contained in this table correspond to figure 2. Coefficient represents difference in standardised parent-report SDQ score between children born between 1st September-31st August. “Unadjusted Model” = Age within School Year entered in the regression alone. “All Covariates” = Model after adjustments for all covariates.

|  | Unadjusted |  |  | All Covariates |  |  |
| --- | --- | --- | --- | --- | --- | --- |
|  | **Coef.** | **95% CI** | **p** | **Coef.** | **95% CI** | **p** |
| 4 Years | -0.02 | [-0.12, 0.08] | 0.674 | 0.01 | [-0.086, 0.106] | 0.84 |
| 7 Years | 0.13 | [0.03, 0.24] | 0.014 | 0.15 | [0.054, 0.254] | <0.01 |
| 8 Years | 0.18 | [0.08, 0.29] | 0.001 | 0.20 | [0.095, 0.302] | <0.001 |
| 9 Years | 0.14 | [0.03, 0.24] | 0.011 | 0.16 | [0.056, 0.260] | <0.01 |
| 11 Years | 0.20 | [0.09, 0.30] | <0.001 | 0.22 | [0.117, 0.323] | <0.001 |
| 13 Years | 0.12 | [0.01, 0.23] | 0.029 | 0.14 | [0.033, 0.243] | 0.01 |
| 16 Years | 0.11 | [-0.01, 0.23] | 0.062 | 0.13 | [0.012, 0.244] | 0.03 |
| 25 Years | 0.03 | [-0.10, 0.16] | 0.668 | 0.04 | [-0.087, 0.168] | 0.53 |

**Table S18.** Regression results for Self-rated SMFQ by relative age, restricted to 4 weeks either side of September 1^st^ cut-off, Imputed data (N=1690) The numbers contained in this table correspond to figure 3. Coefficient represents difference in standardised self-report SMFQ score between children born between 1st September-31st August. “Unadjusted Model” = Age within School Year entered in the regression alone. “All Covariates” = Model after adjustments for all covariates.

|  | Unadjusted |  |  | All Covariates |  |  |
| --- | --- | --- | --- | --- | --- | --- |
|  | **Coef.** | **95% CI** | **p** | **Coef.** | **95% CI** | **P** |
| 10 Years | 0.01 | [-0.11, 0.13] | 0.88 | 0.03 | [-0.09, 0.15] | 0.59 |
| 13 Years | -0.05 | [-0.16, 0.07] | 0.42 | -0.04 | [-0.16, 0.08] | 0.51 |
| 14 Years | 0.09 | [-0.03, 0.20] | 0.16 | 0.08 | [-0.03, 0.20] | 0.16 |
| 16 Years | 0.01 | [-0.12, 0.13] | 0.93 | 0.01 | [-0.11, 0.13] | 0.87 |
| 17 Years | -0.11 | [-0.25, 0.04] | 0.14 | -0.01 | [-0.24, 0.04] | 0.17 |
| 18 Years | -0.09 | [-0.22, 0.04] | 0.19 | -0.01 | [-0.21, 0.05] | 0.24 |
| 21 Years | 0.01 | [-0.13, 0.15] | 0.85 | 0.02 | [-0.12, 0.15] | 0.82 |
| 22 Years | 0.04 | [-0.10, 0.18] | 0.58 | 0.039 | [-0.10, 0.18] | 0.59 |
| 23 Years | 0.01 | [-0.13, 0.14] | 0.92 | 0.020 | [-0.11, 0.15] | 0.77 |
| 25 Years | 0.10 | [-0.04, 0.24] | 0.15 | 0.108 | [-0.03, 0.24] | 0.12 |

**Table S19.** Regression results for parent-rated SMFQ by relative age, restricted to 4 weeks either side of September 1^st^ cut-off, Imputed data (N=1642) The numbers contained in this table correspond to figure 4. Coefficient represents difference in standardised parent-report SMFQ score between children born between 1st September-31st August. “Unadjusted Model” = Age within School Year entered in the regression alone. “All Covariates” = Model after adjustments for all covariates

|  | Unadjusted |  |  | All Covariates |  |  |
| --- | --- | --- | --- | --- | --- | --- |
|  | Coef. | 95% CI | p | Coef. | 95% CI | p |
| 9 Years | 0.03 | [-0.08, 0.15] | 0.56 | 0.05 | [-0.06, 0.17] | 0.36 |
| 11 Years | 0.10 | [-0.02, 0.22] | 0.11 | 0.11 | [-0.01, 0.23] | 0.07 |
| 13 Years | 0.02 | [-0.10, 0.14] | 0.78 | 0.03 | [-0.09, 0.15] | 0.67 |
| 16 Years | -0.01 | [-0.14, 0.12] | 0.83 | -0.02 | [-0.14, 0.11] | 0.80 |

**Table S20.** Regression results for parent-rated SDQ total difficulties scores by relative age, restricted to 8 weeks either side of September 1^st^ cut-off, Imputed data (N=4042) The numbers contained in this table correspond to figure 2. Coefficient represents difference in standardised parent-report SDQ score between children born between 1st September-31st August. “Unadjusted Model” = Age within School Year entered in the regression alone. “All Covariates” = Model after adjustments for all covariates.

|  | Unadjusted |  |  | All Covariates |  |  |
| --- | --- | --- | --- | --- | --- | --- |
|  | Coef. | 95% CI | p | Coef. | 95% CI | p |
| 4 Years | -0.03 | [-0.10, 0.05] | 0.50 | -0.01 | [-0.08, 0.07] | 0.89 |
| 7 Years | 0.13 | [0.05, 0.21] | 0.001 | 0.15 | [0.07, 0.22] | <0.001 |
| 8 Years | 0.18 | [0.10, 0.26] | <0.001 | 0.19 | [0.11, 0.27] | <0.001 |
| 9 Years | 0.15 | [0.07, 0.23] | <0.001 | 0.16 | [0.08, 0.24] | <0.001 |
| 11 Years | 0.21 | [0.13, 0.29] | <0.001 | 0.22 | [0.14, 0.30] | <0.001 |
| 13 Years | 0.14 | [0.05, 0.22] | 0.002 | 0.14 | [0.06, 0.23] | 0.001 |
| 16 Years | 0.10 | [0.00, 0.19] | 0.05 | 0.10 | [0.01, 0.20] | 0.03 |
| 25 Years | -0.00 | [-0.11, 0.11] | 0.99 | 0.01 | [-0.10, 0.12] | 0.91 |

**Table S21**. Regression results for Self-rated SMFQ by relative age, restricted to 8 weeks either side of September 1^st^ cut-off, Imputed data (N=3383) The numbers contained in this table correspond to figure 3. Coefficient represents difference in standardised self-report SMFQ score between children born between 1st September-31st August. “Unadjusted Model” = Age within School Year entered in the regression alone. “All Covariates” = Model after adjustments for all covariates.

|  | Unadjusted |  |  | All Covariates |  |  |
| --- | --- | --- | --- | --- | --- | --- |
|  | **Coef.** | **95% CI** | **p** | **Coef.** | **95% CI** | **p** |
| 10 Years | 0.01 | [-0.08, 0.10] | 0.86 | 0.02 | [-0.07, 0.11] | 0.70 |
| 13 Years | -0.05 | [-0.14, 0.05] | 0.33 | -0.04 | [-0.13, 0.05] | 0.42 |
| 14 Years | 0.08 | [-0.02, 0.17] | 0.11 | 0.08 | [-0.01, 0.17] | 0.09 |
| 16 Years | 0.03 | [-0.07, 0.12] | 0.61 | 0.03 | [-0.07, 0.13] | 0.52 |
| 17 Years | -0.10 | [-0.22, 0.02] | 0.10 | -0.09 | [-0.20, 0.03] | 0.15 |
| 18 Years | -0.08 | [-0.18, 0.02] | 0.13 | -0.07 | [-0.17, 0.03] | 0.18 |
| 21 Years | 0.03 | [-0.09, 0.14] | 0.66 | 0.03 | [-0.08, 0.14] | 0.61 |
| 22 Years | 0.04 | [-0.07, 0.15] | 0.43 | 0.05 | [-0.06, 0.16] | 0.39 |
| 23 Years | -0.01 | [-0.11, 0.10] | 0.90 | 0.01 | [-0.10, 0.11] | 0.93 |
| 25 Years | 0.11 | [-0.00, 0.22] | 0.05 | 0.12 | [0.01, 0.23] | 0.03 |

**Table S22.** Regression results for parent-rated SMFQ by relative age, restricted to 8 weeks either side of September 1^st^ cut-off, Imputed data (N=5297) The numbers contained in this table correspond to figure 4. Coefficient represents difference in standardised parent-report SMFQ score between children born between 1st September-31st August. “Unadjusted Model” = Age within School Year entered in the regression alone. “All Covariates” = Model after adjustments for all covariates

|  | Unadjusted |  |  | All Covariates |  |  |
| --- | --- | --- | --- | --- | --- | --- |
|  | **Coef.** | **95% CI** | **p** | **Coef.** | **95% CI** | **p** |
| 9 Years | 0.07 | [-0.02, 0.15] | 0.13 | 0.07 | [-0.01, 0.16] | 0.09 |
| 11 Years | 0.12 | [0.03, 0.21] | 0.01 | 0.12 | [0.04, 0.21] | 0.01 |
| 13 Years | 0.02 | [-0.07, 0.11] | 0.73 | 0.02 | [-0.07, 0.10] | 0.73 |
| 16 Years | 0.01 | [-0.09, 0.11] | 0.87 | 0.01 | [-0.09, 0.11] | 0.85 |

**Table S23.** Complete-case analysis. Outcome: Parent-Rated SDQ (4-25 years) Independent variable: Relative Age (week of birth). Coefficient represents difference in standardised parent-report SDQ score between children born between 1st September and those born on 31st August (i.e., 1 year difference in age at starting school). “Unadjusted” = Age within School Year entered in the regression alone. “All Covariates” = Model after adjustments for all covariates.

|  |  | Unadjusted | |  |  | All Covariates |  |  |  |
| --- | --- | --- | --- | --- | --- | --- | --- | --- | --- |
| Total Difficulties | | **N** | **Coefficient** | **95% CI** | **p** | **N** | **Coefficient** | **95% CI** | **p** |
|  | **4 Years** | 9312 | 0.02 | [-0.04, 0.09] | 0.512 | 7248 | 0.06 | [-0.01, 0.13] | 0.11 |
|  | **7 Years** | 8281 | 0.15 | [0.08, 0.22] | <0.001 | 6541 | 0.18 | [0.10, 0.25] | <0.001 |
|  | **8 Years** | 7669 | 0.17 | [0.09, 0.22] | <0.001 | 5933 | 0.19 | [0.11, 0.27] | <0.001 |
|  | **9 Years** | 7,934 | 0.15 | [0.08, 0.22] | <0.001 | 6014 | 0.17 | [0.10, 0.25] | <0.001 |
|  | **11 Years** | 7253 | 0.21 | [0.14, 0.28] | <0.001 | 5562 | 0.25 | [0.17, 0.33] | <0.001 |
|  | **13 Years** | 6933 | 0.15 | [0.07, 0.22] | <0.001 | 5320 | 0.15 | [0.06, 0.23] | 0.001 |
|  | **16 Years** | 5554 | 0.07 | [-0.02, 0.16] | 0.108 | 4365 | 0.09 | [0.00, 0.18] | 0.05 |
|  | **25 Years** | 4076 | -0.05 | [-0.15, 0.06] | 0.282 | 3233 | -0.03 | [-0.14, 0.07] | 0.52 |
| Conduct Problems | |  |  |  |  |  |  |  |  |
|  | **4 Years** | 9341 | 0.01 | [-0.06, 0.07] | 0.8 | 7268 | 0.02 | [-0.05, 0.09] | 0.54 |
|  | **7 Years** | 8310 | -0.01 | [-0.08, 0.06] | 0.76 | 6553 | 0 | [-0.07, 0.08] | 0.97 |
|  | **8 Years** | 7682 | 0.03 | [-0.05, 0.09] | 0.48 | 5940 | 0.06 | [-0.02, 0.14] | 0.13 |
|  | **9 Years** | 7,962 | -0.02 | [-0.09, 0.04] | 0.53 | 6032 | 0 | [-0.08, 0.08] | 1 |
|  | **11 Years** | 7,256 | 0.07 | [<0.01, 0.15] | 0.05 | 5561 | 0.09 | [0.01, 0.17] | 0.03 |
|  | **13 Years** | 6958 | 0.02 | [-0.06, 0.09] | 0.69 | 5338 | 0.03 | [-0.05, 0.11] | 0.49 |
|  | **16 Years** | 5598 | -0.01 | [-0.09 ,0.08] | 0.86 | 4395 | 0.01 | [-0.08, 0.10] | 0.81 |
|  | **25 Years** | 4290 | -0.01 | [-0.1, 0.1] | 0.88 | 3406 | 0.02 | [-0.09, 0.12] | 0.74 |
| Emotional problems | |  |  |  |  |  |  |  |  |
|  | **4 Years** | 9355 | 0.09 | [0.02, 0.15] | 0.01 | 7271 | 0.09 | [0.02, 0.17] | 0.01 |
|  | **7 Years** | 8300 | 0.13 | [0.06, 0.2] | <0.01 | 6552 | 0.14 | [0.07, 0.22] | <0.001 |
|  | **8 Years** | 7679 | 0.09 | [0.02, 0.14] | 0.02 | 5940 | 0.1 | [0.02, 0.18] | 0.01 |
|  | **9 Years** | 7946 | 0.11 | [0.04, 0.18] | <0.01 | 6026 | 0.12 | [0.04, 0.20] | <0.01 |
|  | **11 Years** | 7239 | 0.1 | [0.03, 0.18] | 0.01 | 5554 | 0.14 | [0.05, 0.22] | <0.01 |
|  | **13 Years** | 6961 | 0.08 | [<0.01, 0.16] | 0.04 | 5344 | 0.07 | [-0.01, 0.16] | 0.1 |
|  | **16 Years** | 5587 | 0.09 | [0.01, 0.18] | 0.03 | 4390 | 0.09 | [0.00, 0.18] | 0.04 |
|  | **25 Years** | 4299 | -0.05 | [-0.15, 0.04] | 0.28 | 3407 | -0.03 | [-0.14, 0.07] | 0.51 |
| Hyperactivity Problems | | |  |  |  |  |  |  |  |
|  | **4 Years** | 9347 | <0.01 | [-0.06, 0.07] | 0.897 | 7269 | 0.06 | [-0.01, 0.13] | 0.1 |
|  | **7 Years** | 8286 | 0.17 | [0.10, 0.24] | <0.01 | 6548 | 0.22 | [0.15, 0.30] | <0.001 |
|  | **8 Years** | 7680 | 0.22 | [0.14, 0.28] | <0.01 | 5936 | 0.24 | [0.16, 0.32] | <0.001 |
|  | **9 Years** | 7963 | 0.19 | [0.12, 0.26] | <0.01 | 6037 | 0.21 | [0.14, 0.29] | <0.001 |
|  | **11 Years** | 7238 | 0.26 | [0.19, 0.33] | <0.01 | 5555 | 0.29 | [0.21, 0.37] | <0.001 |
|  | **13 Years** | 6958 | 0.18 | [0.11, 0.25] | <0.01 | 5340 | 0.19 | [0.11, 0.28] | <0.001 |
|  | **16 Years** | 5597 | 0.06 | [-0.03, 0.14] | 0.175 | 4396 | 0.09 | [0.00, 0.18] | 0.05 |
|  | **25 Years** | 4294 | -0.02 | [-0.12, 0.08] | 0.677 | 3406 | -0.03 | [-0.13, 0.07] | 0.55 |
| Peer Problems | |  |  |  |  |  |  |  |  |
|  | **4 Years** | 9354 | -0.03 | [-0.09, 0.04] | 0.42 | 7272 | -0.03 | [-0.10, 0.04] | 0.4 |
|  | **7 Years** | 8305 | 0.07 | [<0.01, 0.14] | 0.06 | 6549 | 0.07 | [-0.01, 0.14] | 0.08 |
|  | **8 Years** | 7678 | 0.08 | [0.01, 0.13] | 0.03 | 5937 | 0.1 | [0.02, 0.18] | 0.01 |
|  | **9 Years** | 7952 | 0.13 | [0.06, 0.2] | <0.01 | 6025 | 0.14 | [0.06, 0.22] | <0.01 |
|  | **11 Years** | 7261 | 0.12 | [0.04, 0.19] | <0.01 | 5564 | 0.15 | [0.07, 0.23] | <0.001 |
|  | **13 Years** | 6958 | 0.11 | [0.03, 0.18] | 0.01 | 5340 | 0.1 | [0.02, 0.19] | 0.02 |
|  | **16 Years** | 5589 | 0.02 | [-0.07, 0.11] | 0.69 | 4387 | 0.03 | [-0.07, 0.12] | 0.58 |
|  | **25 Years** | 4278 | -0.06 | [-0.16, 0.05] | 0.23 | 3394 | -0.04 | [-0.15, 0.06] | 0.42 |

**Table S24.** Complete-case analysis: Self-Rated SMFQ (10-25 years) Independent variable: Relative Age (week of birth), Coefficient represents difference in standardised self-report SMFQ score between children born between 1st September-31st August. “Unadjusted” = Age within School Year entered in the regression alone. “All Covariates” = Model after adjustments for all covariates.

| **Unadjusted** | |  |  |  | **All covariates** | |  |  |
| --- | --- | --- | --- | --- | --- | --- | --- | --- |
| **Age** | N | Coefficient | [95% CI] | p | N | Coefficient | [95% CI] | p |
| **10 Years** | 7245 | 0.02 | [-0.05, 0.10] | 0.58 | 5398 | 0.03 | [-0.06, 0.11] | 0.53 |
| **13 Years** | 6607 | <.01 | [-0.08, 0.08] | 0.99 | 4935 | 0.01 | [-0.08, 0.10] | 0.83 |
| **14 Years** | 5925 | 0.11 | [0.02, 0.19] | 0.01 | 4493 | 0.11 | [0.02, 0.21] | 0.02 |
| **16 Years** | 4939 | 0.08 | [-0.01, 0.17] | 0.08 | 3780 | 0.12 | [0.02, 0.22] | 0.02 |
| **17 Years** | 3299 | -0.09 | [-0.2, 0.02] | 0.11 | 2534 | -0.09 | [-0.21, 0.03]] | 0.15 |
| **18 Years** | 4444 | -0.03 | [-0.13, 0.06] | 0.48 | 3357 | 0 | [-0.10, 0.11] | 0.96 |
| **21 Years** | 3271 | 0.02 | [-0.1, 0.13] | 0.79 | 2506 | 0.03 | [-0.09, 0.15] | 0.63 |
| **22 Years** | 3869 | 0.08 | [-0.03, 0.18] | 0.15 | 2880 | 0.06 | [-0.06, 0.18] | 0.31 |
| **23 Years** | 3972 | -0.02 | [-0.12, 0.08] | 0.72 | 2904 | -0.03 | [-0.15, 0.08] | 0.57 |
| **25 Years** | 3962 | 0.12 | [0.02, 0.22] | 0.02 | 2882 | 0.09 | [-0.02, 0.20] | 0.12 |

**Table S25.** Complete-case analysis– outcome: Parent-Rated SMFQ (9-16 years) Independent variable: Relative Age (week of birth), Coefficient represents difference in standardised parent-report SMFQ score between children born between 1st September-31st August. “Unadjusted” = Age within School Year entered in the regression alone. “All Covariates” = Model after adjustments for all covariates.

| **Unadjusted** | |  |  |  | **All Covariates** | |  |  |
| --- | --- | --- | --- | --- | --- | --- | --- | --- |
| **Age** | **N** | **Coefficient** | **[95% CI]** | **P** | **N** | **Coefficient** | **[95% CI]** | **P** |
| **9 Years** | 7966 | 0.13 | [0.05, 0.20] | <0.01 | 6036 | 0.14 | [0.07, 0.22] | <.001 |
| **11 Years** | 7201 | 0.17 | [0.10, 0.25] | <0.01 | 5526 | 0.2 | [0.12, 0.28] | <.001 |
| **13 Years** | 6899 | 0.04 | [-0.03, 0.12] | 0.25 | 5295 | 0.07 | [-0.02, 0.15] | 0.12 |
| **16 Years** | 5383 | 0.05 | [-0.03, 0.14] | 0.21 | 4224 | 0.1 | [0.01, 0.20] | 0.04 |

**Table S26.** Generalized estimating equation (GEE) results for parent-rated SDQ total difficulties (N=11116). Coefficient represents difference in standardised parent-report SDQ score between children born between 1st September-31st August.

|  | Coef. | p | 95% CI |
| --- | --- | --- | --- |
| 4 Years | 0.02 | 0.63 | [-0.05, 0.08] |
| 7 Years | 0.13 | <0.001 | [0.07, 0.20] |
| 8 Years | 0.17 | <0.001 | [0.10, 0.23] |
| 9 Years | 0.15 | <0.001 | [0.08, 0.21] |
| 11 Years | 0.20 | <0.001 | [0.14, 0.27] |
| 13 Years | 0.12 | <0.001 | [0.06, 0.19] |
| 16 Years | 0.06 | 0.08 | [-0.01, 0.13] |
| 25 Years | -0.04 | 0.37 | [-0.12, 0.04] |

**Table S27.** Generalized estimating equation (GEE) results for self-rated SMFQ scores (N=9468). Coefficient represents difference in standardised self-report SMFQ score between children born between 1st September-31st August.

|  | Coef. | p | 95% CI |
| --- | --- | --- | --- |
| 10 years | 0.02 | 0.63 | [-0.06, 0.09] |
| 13 years | -0.01 | 0.88 | -0.09, 0.08] |
| 14 years | 0.10 | 0.03 | [0.01, 0.19] |
| 16 years | 0.04 | 0.42 | [-0.06, 0.14] |
| 17 years | -0.10 | 0.06 | [-0.21, 0.01] |
| 18 years | -0.05 | 0.31 | [-0.15, 0.05] |
| 21 years | -0.01 | 0.83 | [-0.12, 0.10] |
| 22 years | 0.03 | 0.57 | [-0.07, 0.14] |
| 23 years | -0.02 | 0.77 | [-0.12, 0.09] |
| 25 years | 0.11 | 0.04 | [0.01, 0.22] |

**Table S28.** Generalized estimating equation (GEE) results for parent-rated SMFQ scores (N=9164). Coefficient represents difference in standardised parent-report SMFQ score between children born between 1st September-31st August.

|  | Coef. | p | 95% CI |
| --- | --- | --- | --- |
| 9 years | 0.12 | 0.00 | [0.05, 0.19] |
| 11 years | 0.04 | 0.32 | -[0.04, 0.12] |
| 13 years | -0.08 | 0.04 | -[0.16, 0.00] |
| 16 years | -0.08 | 0.09 | [-0.16, 0.01] |
